# Protein profiling and network enrichment analysis in individuals before and after the onset of rheumatoid arthritis

**DOI:** 10.1186/s13075-019-2066-9

**Published:** 2019-12-16

**Authors:** Mikael Brink, Anders Lundquist, Andrey Alexeyenko, Kristina Lejon, Solbritt Rantapää-Dahlqvist

**Affiliations:** 1 Department of Public Health and Clinical Medicine, Rheumatology, Umeå University, 901 87 Umeå, Sweden; 2 Department of Clinical Microbiology, Division of Infection and Immunology, Umeå University, 901 87 Umeå, Sweden; 3 Science for Life Laboratory, Department of Microbiology, Tumor and Cell Biology (MTC), Karolinska Institutet, Stockholm, Sweden; 4 Division of Infection and Immunology, Department of Clinical Microbiology, Umeå University, Umeå, Sweden

**Keywords:** Rheumatoid arthritis, Pre-symptomatic stage, Protein levels, Plasma biomarkers

## Abstract

**Background:**

Antibodies and upregulated cytokines and chemokines predate the onset of rheumatoid arthritis (RA) symptoms. We aimed to identify the pathways related to the early processes leading to RA development, as well as potential novel biomarkers, using multiple protein analyses.

**Methods:**

A case-control study was conducted within the Biobank of northern Sweden. The plasma samples from 118 pre-symptomatic individuals (207 samples; median predating time 4.1 years), 79 early RA patients, and 74 matched controls were analyzed. The levels of 122 unique proteins with an acknowledged relationship to autoimmunity were analyzed using 153 antibodies and a bead-based multiplex system (FlexMap3D; Luminex Corp.). The data were analyzed using multifactorial linear regression model, random forest, and network enrichment analysis (NEA) based on the 10 most significantly differentially expressed proteins for each two-by-two group comparison, using the MSigDB collection of hallmarks.

**Results:**

There was a high agreement between the different statistical methods to identify the most significant proteins. The adipogenesis and interferon alpha response hallmarks differentiated pre-symptomatic individuals from controls. These two hallmarks included proteins involved in innate immunity. Between pre-symptomatic individuals and RA patients, three hallmarks were identified as follows: apical junction, epithelial mesenchymal transition, and TGF-β signaling, including proteins suggestive of cell interaction, remodulation, and fibrosis. The adipogenesis and heme metabolism hallmarks differentiated RA patients from controls.

**Conclusions:**

We confirm the importance of interferon alpha signaling and lipids in the early phases of RA development. Network enrichment analysis provides a tool for a deeper understanding of molecules involved at different phases of the disease progression.

## Introduction

Rheumatoid arthritis (RA) is a common autoimmune disease, characterized by immune cell infiltration of the joints, cartilage and bone degradation, and resulting in joint pain and stiffness. The etiopathogenic process leading to disease development and progression is not completely understood, although there are autoimmune processes ongoing long before any clinical symptoms have occurred—i.e., an increased amount of anti-citrullinated peptide antibodies (ACPA) and/or rheumatoid factor (RF) [1–5]. During the pre-symptomatic period of the disease, a gradual broadening of the autoantibody repertoire is observed closer to disease onset, i.e., epitope spreading [4, 6, 7]. Additionally, elevated levels of cytokines and chemokines have been shown in the plasma from pre-symptomatic individuals sampled years before any symptoms or clinical manifestations are present compared with controls [8, 9]. Also, using the expression levels of type I interferon-related genes has been shown to be elevated in both seropositive at-risk individuals and in pre-symptomatic individuals compared to controls [10]. In this study, analyzing a larger set of protein markers than previous studies, we aimed to gain further insight into which molecular processes are involved in RA development prior to the onset of symptoms and to identify potential early biomarkers.

## Material and methods

### Subjects

A case-control study was conducted with individuals included in population surveys within the Medical Biobank of Northern Sweden. The criteria for the recruitment, collection, and storage of the blood samples have been described in detail previously [3]. The cohorts included in the Medical Biobank are population-based, and all adult individuals residing in the county of Västerbotten are continuously invited to participate. To identify individuals who donated blood samples prior to the onset of RA symptoms, the registers at the Medical Biobank were coanalyzed with those of patients with RA, fulfilling the 1987 American Rheumatism Association classification criteria for RA [11] at the Department of Rheumatology, University Hospital, Umeå, and with a known date for the onset of symptoms. In this study, 368 samples were included for protein analysis: 209 samples from 118 pre-symptomatic individuals, 85 samples from these individuals after they were diagnosed with RA, and 74 samples from controls matched to the pre-symptomatic individuals (Additional file 1: Table S1). One sample from the RA patients was excluded due to duplication. Seven samples were excluded due to failure in the analysis procedure and/or outlying data (5 samples from RA patients and 2 from pre-symptomatic individuals, all from different individuals). Consequently, 118 individuals, referred to as pre-symptomatic individuals, who had donated 207 blood samples at different time points before the onset of symptoms were included in this study. Of the 118 individuals, 60 contributed to 1 sample, 32 contributed to 2 samples, 21 contributed to 3 samples, and 5 individuals contributed to 4 samples. Of these 118 individuals, 79 were also sampled at the time of diagnosis—i.e., hereafter referred to as RA patients. The median [interquartile range (IQR)] time predating the onset of symptoms including all 207 samples was 4.1 (4.2) years. Control subjects were identified from the same cohorts within the registers of the Medical Biobank of northern Sweden and were matched for age, sex, and date of blood sampling; from them, 74 were randomly selected. All samples were thawed when dispensed onto 96-well plates and were thereafter refrozen until analysis.

### Protein analysis

Using antibody bead arrays, 184 Human Protein Atlas (HPA) antibodies were employed to target 122 unique proteins selected based on presumed relationships to inflammation, immune response, and soft tissue, and availability of target antibodies ([8, 12–15] and Personal Communication). Matched pairs (from pre-symptomatic individuals, RA patients, and controls) of samples were randomized within the same plate of the 4 96-well plates, sample replicates were added to each plate, and assays were performed twice using newly prepared (labeled) samples. The samples were analyzed using a direct protein-labeling approach detailed elsewhere [16]. Briefly, the plasma samples were diluted and biotinylated. Antibodies were coupled to beads, and all individual bead IDs were combined to create a bead array. The labeled samples were then heat treated and combined with the bead array for analysis. After washing the beads and detection, the analysis of the captured protein abundance occurred in a flow cytometer system (FlexMap3D; Luminex Corp., Austin, TX, USA). The median fluorescence intensity (MFI) of at least 32 beads per antibody was chosen for data analysis.

The sample-by-sample variation within each assay plate was considered with the probabilistic quotient normalization (PQN) [17]. PQN accounts for the differences in the antibody dynamics by adjusting for the normalizing factor using antibody-specific weights that equal to 1 (correlation with the normalization factor) (Dodig-Crnkovic et al., unpublished). To overcome plate effects, we adjusted using Multi-MA [18].

Robust PCA was used to filter for outlying samples. Failed and outlying sample data were reported as NA. After quality control, i.e., only antibodies with values of Spearman correlation between two replicated assays > 0.5, 31 proteins were excluded from further analysis, leaving 153 HPA antibodies and 107 unique proteins for further analysis (Additional file 2: Table S2).

### Statistics

The protein expression data were primarily normalized as described in the “Protein analysis” section. To make the protein profiles amenable to parametric statistical methods, we further rendered them to log values. Protein expression was tested for each *i* of the 153 antibodies in a multifactorial linear regression model of the form aov(expr[,*i*] ~ Case + TTS + Error (ID)) (using R syntax), where “TTS” (time to symptom in months) was interpreted as a quantitative main factor, while the “Case” (0, control; 1, pre-symptomatic; 2, RA) was estimated as an ordered main factor. The patients (ID) served as replicates in this model. Differentially expressed (DE) proteins were thus identified by the significance of the “Case” *p* values.

Using this model, we performed three comparisons of interest: (a) contrast between controls and the pre-symptomatic state, (b) contrast between the pre-symptomatic and RA states, and (c) contrast between the RA and control state. For further network analysis, we needed to characterize each of these comparisons with protein lists of equal length. This resulted in producing in lists of ten most differentially expressed proteins (ranked by “Case” *p* values) in each of the three analyses above, regardless of the formal significance of individual proteins. *p* value levels of significance after adjustment for multiple testing (by Benjamini-Hochberg) are reported in Additional file 2: Table S2.

### Network enrichment analysis

Biological phenomena can be characterized at the molecular level via pathway enrichment analysis. Among the multiple existing versions of the latter, we chose the method of network enrichment analysis (NEA) [19]. NEA can analyze differentially expressed protein lists (i.e., altered gene sets (AGS)) in the way most similar to that of overrepresentation analysis (ORA) [20]. The major difference between NEA and the network-free alternatives—ORA and most of the other methods—is that the former accounts for and evaluates enrichment significance via the number of network edges (links that characterize protein functional coupling via different molecular mechanisms [21]) between any proteins of AGS (i.e., the list in question) and a pathway list (referred as a functional gene set (FGS)). Due to the high density of edges currently known in the global network (the median is ~ 50 to 100 per protein node), NEA possesses a very high statistical power to detect enrichment (even in shorter lists such as *N* = 10) and is more robust when validated across independent datasets [22]. Another advantage is that NEA incorporates pathway proteins that themselves may not change expression, although they could enable, for example, transcriptional regulation, phosphorylation, or decay of the studied experimental proteins.

For the global network in NEA, we used the functional links from several curated databases collected in the Pathway Commons project (version 9) [23] with 846,631 links among 20,063 unique human proteins. The NEA algorithm ignores confidence or other attributes of the network links, which was a relevant feature in this analysis, since the Pathway Commons network collected highly confident by rather heterogeneous links, based on different analytical scales across a number of database projects.

For the FGSs (pathways), the MSigDB collection of hallmarks was employed [24]. It contained 50 protein sets compiled to provide maximal coverage of the most important cellular processes with a minimal overlap between the protein members of different hallmarks. The analysis was run in R environment using package NEArender of version 1.4 (19). NEArender produced *p* values of network enrichment for each AGS-FGS pair. The latter were adjusted for multiple testing by Bonferroni correction, i.e., *p* (Bonferroni) = *p* (NEA) × *N*_hallmarks_ (Benjamini-Hochberg correction would be less suitable due to the low number *N*_hallmarks_ = 50).

### Differential enrichment

One specific feature of the present analysis was in profiling a predefined set of proteins with either a known or suggested relationship to immunity and RA. In this context, any enrichment method would identify multiple FGSs relevant to these functional focuses. Therefore, in addition to the standard NEA run on the actual protein AGSs as described above, we implemented a control permutation test. More specifically, for each of the six experimental AGSs, we generated 10,000 sets of the same size, sampled with replacement from the total pool of the 153 antibodies. Next, for each FGS hallmark with a significant NEA score, we required that the permutation *p* value from the latter test did not exceed 0.05. In other words, an observation that an AGS list *X* was enriched in connections with an FGS hallmark *Y* should not have been recapitulated in more than 5% of the random tests of *X*_*Ri*_ vs. *Y*, where *i*⊂{1 … 10,000}. Hence, the permutation *p* value reported the probability of the null hypothesis, namely that enrichment is due to the functional focus of all the selected 153 proteins rather than a particular experimental AGS. This filtering enabled selecting hallmarks specifically pertinent to our analysis.

### Random forest analysis

Three separate classification models to classify pre-symptomatic individuals vs. controls, RA patients vs. controls, and pre-symptomatic individuals vs. RA patients were applied. We used random forests [25] as implemented in the package *randomForest* [26] version 4.6-14 in the R software [27], version 3.5.0. To estimate class membership probabilities, we used out-of-bag estimation (which is the default setting) to obtain valid estimates of the relevant probabilities.

The error rates used for estimating the AUC are the out-of-bag (OOB) estimates provided by the RandomForest package. The OOB estimates yield a quite good approximation to external validation, for details, see, e.g., [28].

## Results

### Linear model analysis

Applying multifactorial modeling, the pairs of the experimental groups were compared (factor “Case”; controls, pre-symptomatic individuals, or RA patients) and included the analyzed 153 protein antibodies (representing 107 unique proteins). For the individuals who had consecutive pre-symptomatic samples available, the linear model of protein expression (PE) also accounted for sampling order and, more precisely, time in months before the RA diagnosis (factor TTS); available replicates over same individuals were used to estimate residual error: PE = *β*_*c*_Case + *β*_*t*_TTS + *ε*(individual).

In these analyses, the levels of 78 (62 unique) proteins were found to be significantly different (*p* value for “Case”) between pre-symptomatic individuals and controls, 121 (88 unique) differed between RA patients and controls, and 49 (45 unique) proteins differed in comparison between pre-symptomatic individuals and RA patients (before adjustments for multiple testing). The 10 proteins with the lowest values for each comparison are presented in Table 1. The corresponding numbers of proteins after adjustment for multiple testing were 22 (20 unique), 93 (75 unique), and 1 protein, respectively. We also considered more complex models with sex and age at the time of sampling as covariates. However, these adjustments, while introducing potential imbalance to the multifactorial linear model, did not affect our results, except for the comparison between patients vs. pre-symptomatic individuals where the TGFB3 protein was not included in the respective AGS (the *p* values in the lineal models increased from 0.004 to 0.0558).

**Table 1:** The ten proteins with the highest significance using multifactorial linear regression for pre-symptomatic individuals, RA patients, and controls compared two-by-two

Pre-symptomatic individuals vs. controls		
Protein	*p* value	Up or downregulated^a^
TNF	1.94E−07	↑
PRR16	2.68E−07	↑
CSF2	2.05E−06	↑
CCDC85C	2.91E−06	↑
CASP8	3.72E−06	↑
IL33^†^	5.45E−06	↑
FAM81A	5.77E−06	↑
SELE	8.44E−06	↑
HTRA1	1.39E−05	↑
MMP10	2.16E−05	↑
Patients vs. controls		
Protein	*p* value	Up or downregulated^a^
TNF	5.52E−26	↑
PRR16	9.82E−26	↑
S100A12	1.06E−24	↑
CSF2	3.33E−24	↑
CASP8	2.35E−23	↑
FAM81A	6.74E−22	↑
MMP10	1.56E−21	↑
HTRA1	2.05E−20	↑
SELE	2.30E−20	↑
ORM1, ORM2^†^	5.80E−20	↑
Pre-symptomatic individuals vs. patients		
Protein	*p* value	Up or downregulated^b^
KCNB2^†^	2.92E−04	↓
S100A12	7.41E−04	↑
EPB41L5^†^	1.97E−03	↑
COL6A1	2.55E−03	↓
ZNF618^†^	3.82E−03	↑
S100A12	4.32E−03	↑
TGFB3^†^	4.43E−03	↓
CCDC85C	6.28E−03	↑
CSF2	6.73E−03	↑
DSC3^†^	6.90E−03	↑
SLC11A1^†^	8.33E−03	↑

^†^Protein included uniquely in one of three top ten protein lists. An expression change based comparison in ^a^pre-symptomatic individuals or RA patients vs. controls and ^b^RA patients vs. pre-symptomatic individuals

*CASP8* caspase 8; *CCDC85C* coiled-coil domain containing 85C; *COL6A1* collagen type VI alpha 1 chain; *CSF2* colony-stimulating factor 2; *DSC3* desmocollin 3; *EPB41L5* erythrocyte membrane protein band 4.1 like 5; *FAM81A* family with sequence similarity 81 member A; *HTRA1* HtrA serine peptidase 1; *IL33* interleukin 33; *KCNB2* potassium voltage-gated channel subfamily B member 2; *MMP10* matrix metallopeptidase 10; *ORM1, ORM2* orosomucoid 1, orosomucoid 2; *PRR16* proline rich 16; *S100A12* S100 calcium-binding protein A12; *SELE* selectin E; *SLC11A1* solute carrier family 11 member 1; *TGFB3* transforming growth factor beta 3; *TNF* tumor necrosis factor; *ZNF618* zinc finger protein 618

### Random forest analyses

**Fig. 1:**
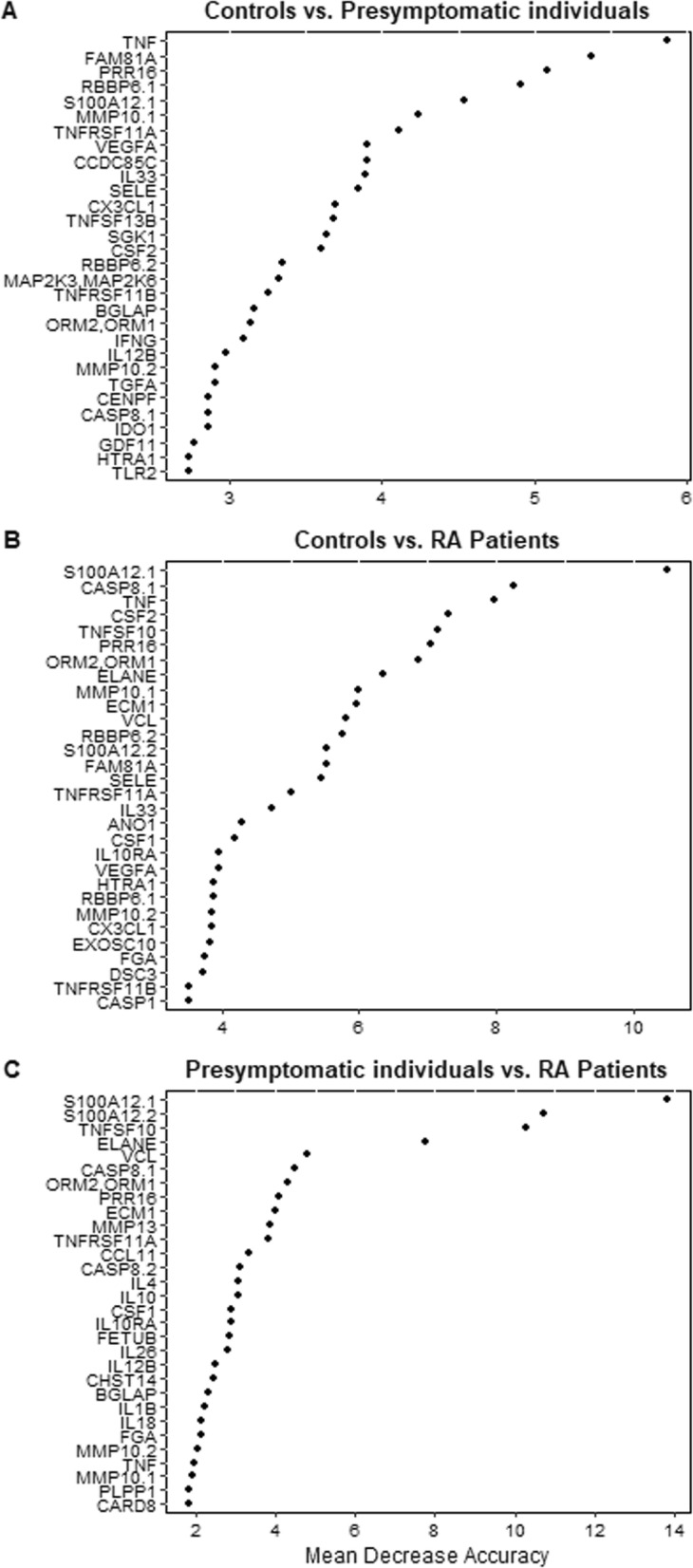
Analysis of the included 153 proteins using random forest analysis, showing the 30 proteins with the highest discriminative ability and corresponding mean decrease accuracy—i.e., the decrease in model accuracy from permuting the values in each feature. Three comparisons were made between **a** controls vs. pre-symptomatic individuals, **b** controls vs. RA patients, and **c** pre-symptomatic individuals and RA patients

The random forest modeling included all 153 proteins. The analysis showed the order of the proteins in terms of their accuracy for discriminating between the compared groups (i.e., their relative importance). The proteins for discriminating pre-symptomatic individuals from controls yielded an area under the curve (AUC) of 0.75 calculated on all proteins. In Fig. 1a, the 30 most important proteins are presented in consecutive order of importance. The AUC comparing pre-symptomatic individuals and RA patients was 0.80, a value expectedly much higher comparing RA patients and controls (AUC = 0.93) (Fig. 1b, c). The discrimination of the groups using the random forest is visualized in Additional file 3: Figure S1.

### Comparison of the results from random forest analysis and linear models

Of the 30 proteins with the highest discriminatory capacity, 27 were significantly different in pre-symptomatic individuals compared with controls analyzed using via linear modeling (*p* < 1.9E−7 to *p* < 0.05). Between RA patients and controls, 29 of the 30 proteins with the highest accuracy for discrimination using random forest analysis were also significantly different using linear modeling (*p* < 5.5E−26 to 1.7E−4). Furthermore, comparing the pre-symptomatic individuals vs. RA patients, 29 of the 30 proteins with the highest discriminatory capacity according to the random forest analysis were significantly different using linear modeling (*p* < 7.4E−4 to *p* < 0.05).

### Gene sets identified by network enrichment analysis

The 10 proteins with the lowest significance from each two-by-two group (controls, pre-symptomatic individuals or RA patients) comparisons were analyzed as AGS in NEA against the hallmark gene set collection (Table 2). In this and the 2 subsequent analyses, we ensured that these proteins did not represent the whole 153-antibody panel by chance. For this purpose, we estimated *differential enrichment* in a random permutation test, upon which only the differentially enriched AGS-FGS cases were reported. Taking this into account, 6 different hallmarks were identified as both significant (Bonferroni-adjusted NEA *p* value < 10^−5^) and specific (differential enrichment *p* value < 0.05): adipogenesis, interferon alpha (IFN-α) response, heme metabolism, apical junction, epithelial-mesenchymal transition, and transforming growth factor-beta (TGF-β) signaling (Table 2). From these analyses, the same collection of proteins was found in 2 different hallmarks (adipogenesis and heme metabolism), for example, for RA patients vs. controls. Furthermore, 3 of the included proteins could not be linked to any of the 50 hallmark gene sets (proline rich 16 [PRR16], coiled-coil domain-containing 85C [CCDC85C], and solute carrier family 11 member 1 [SLC11A1]).

**Table 2:** Ten proteins with the highest significance in multifactorial linear regression (constituting AGS) and presence of a link to the respective hallmark gene sets for pre-symptomatic individuals, RA patients, and controls compared two-by-two

Pre-symptomatic individuals vs. controls							
Protein	Up- or downregulated^a^	Adipogenesis		Interferon alpha response			
		3.74E−09^#^	0.0439^#^	2.08E−07^#^	0.0371^#^		
TNF	↑	+		+			
PRR16	↑						
CSF2	↑	+		+			
CCDC85C	↑						
CASP8	↑	+		+			
IL33^†^	↑			+			
FAM81A	↑	+					
SELE	↑	+		+			
HTRA1	↑	+		+			
MMP10	↑	+					
Patients vs. Controls							
Protein	Up- or downregulated^a^	Adipogenesis		Heme metabolism			
		2.57E−13^#^	0.0058^#^	1.77E−10^#^	0.0022^#^		
TNF	↑	+		+			
PRR16	↑						
S100A12	↑	+		+			
CSF2	↑	+		+			
CASP8	↑	+		+			
FAM81A	↑	+		+			
MMP10	↑	+		+			
HTRA1	↑	+		+			
SELE	↑	+		+			
ORM1, ORM2^†^	↑	+		+			
Pre-symptomatic individuals vs. patients							
Protein	Up- or downregulated^b^	Apical junction		Epithelial-mesenchymal transition		TGF-β signaling	
		8.31E−13^#^	0.0391^#^	9.93E−43^#^	0.0287^#^	1.44E−09^#^	0.0279^#^
KCNB2^†^	↓	+					
S100A12	↑	+		+			
EPB41L5^†^	↑	+		+		+	
COL6A1	↓	+		+		+	
ZNF618^†^	↑					+	
S100A12	↑	+		+			
TGFB3^†^	↓	+		+		+	
CCDC85C	↑	+				+	
CSF2	↑	+		+		+	
DSC3^†^	↑	+		+		+	
SLC11A1^†^	↑						

#Numbers below each hallmark title display enrichment *p* values: general NEA (left) and differential (right)

An expression change-based comparison in ^a^pre-symptomatic individuals or RA patients vs. controls and ^b^RA patients vs. pre-symptomatic individuals

*CASP8* caspase 8; *CCDC85C* coiled-coil domain containing 85C; *COL6A1* collagen type VI alpha 1 chain; *CSF2* colony-stimulating factor 2; *DSC3* desmocollin 3; *EPB41L5* erythrocyte membrane protein band 4.1 like 5; *FAM81A* family with sequence similarity 81 member A; *HTRA1* HtrA serine peptidase 1; *IL33* interleukin 33; *KCNB2* potassium voltage-gated channel subfamily B member 2; *MMP10* matrix metallopeptidase 10; *ORM1, ORM2* orosomucoid 1,orosomucoid 2; *PRR16* proline rich 16; *S100A12* S100 calcium-binding protein A12; *SELE* selectin E; *SLC11A1* solute carrier family 11 member 1; *TGFB3* transforming growth factor beta 3; *TNF* tumor necrosis factor; *ZNF618* zinc finger protein 618

^†^Protein included uniquely in one of three top ten protein lists

### Functional gene sets identified between pre-symptomatic individuals and controls

**Fig. 2:**
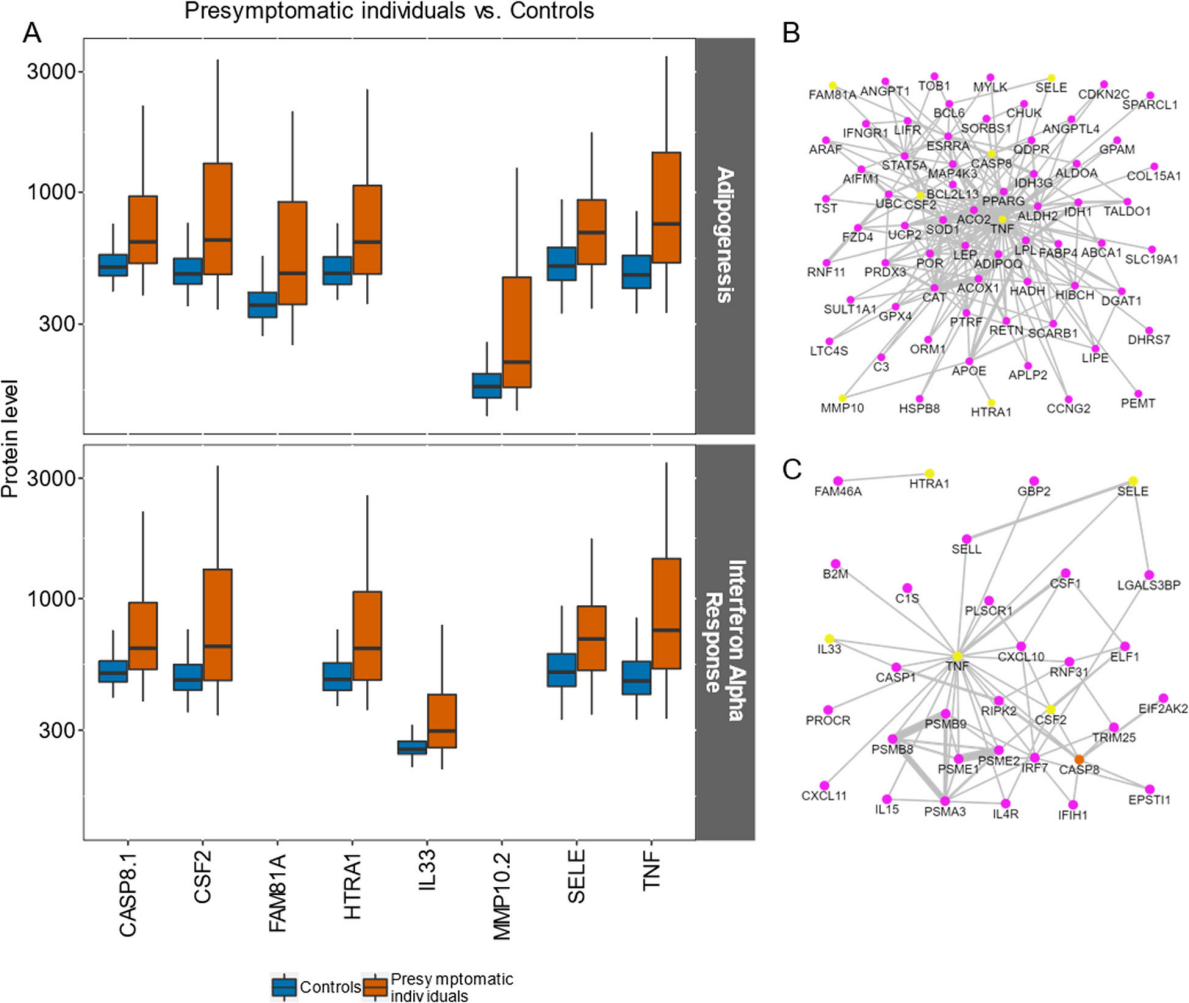
The proteins behind the enrichment toward the hallmark gene sets adipogenesis and interferon alpha in pre-symptomatic individuals and controls. **a** Protein abundance levels (log_10_) in controls vs. pre-symptomatic individuals; the upper panel shows proteins are related to adipogenesis, while the lower panel relates to the interferon alpha gene set. Boundaries of the box indicating Q1 to Q3, vertical black line as the median and whiskers according to Tukey. **b**, **c** Subnetworks extracted from the global Pathway Commons network (version 9, Cerami et al. [23]): links connecting AGS proteins (yellow) with proteins of **b** adipogenesis and **c** interferon alpha FGSs. The orange color indicates proteins found in both AGS and FGS. This and subsequent analyses were performed on the online NEA resource evinet.org [29]. Confidence of the network links (= line thickness) was ignored by the NEA algorithm

In the NEA comparing pre-symptomatic individuals and controls, two hallmark functional gene sets (FGSs) were identified, IFN-α response and adipogenesis. In the IFN-α response gene set, tumor necrosis factor (TNF), caspase 8 (CASP8), colony-stimulating factor 2 (CSF2), interleukin 33 (IL33), HtrA serine peptidase 1 (HTRA1), and selectin E (SELE) contributed to the enriched connectivity with AGS, all with elevated levels in pre-symptomatic individuals (Fig. 2a). CASP8 was also found to contribute to the FGS (Fig. 2c). In the second identified gene set, adipogenesis, TNF, CASP8, CSF2, SELE, HTRA1, matrix metalloproteinase 10 (MMP10), and family with sequence similarity 81 member A (FAM81A) were identified, also showing higher protein levels in pre-symptomatic individuals (Fig. 2a, and Additional file 4: Figure S2). The AGS and FGS proteins included in this gene set are presented in Fig. 2b.

### Gene sets identified between RA patients and controls

**Fig. 3:**
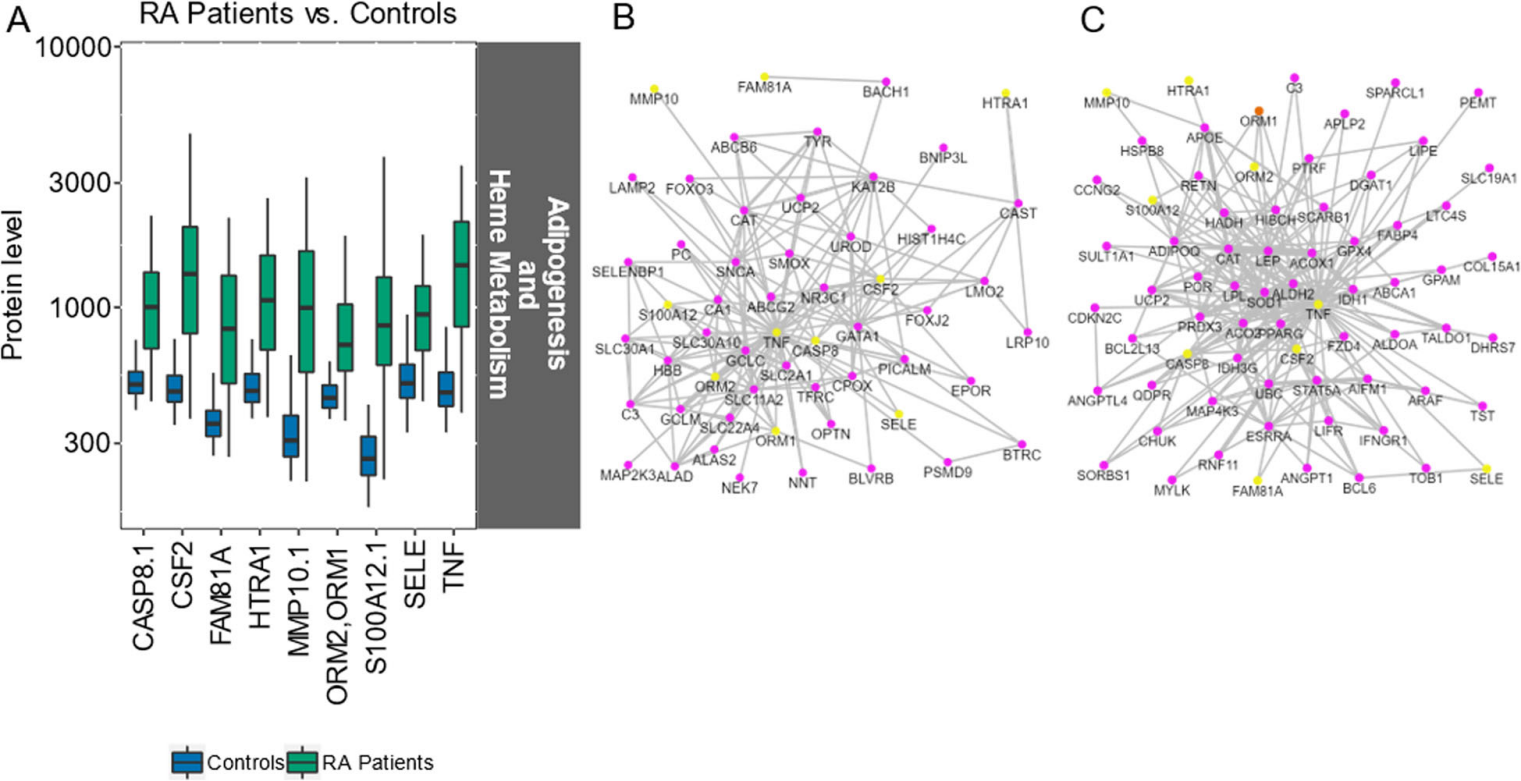
The AGS proteins identified in the hallmark gene sets adipogenesis and heme metabolism in RA patients and controls. **a** Protein abundance levels (log10) in controls and RA patients (the same nine proteins were identified in both gene sets). Boundaries of the box indicating Q1 to Q3, vertical black line as the median and whiskers according to Tukey. **b**, **c** Subnetworks extracted from the global Pathway Commons network: links connecting AGS proteins (yellow) and AGS and FGS proteins (orange) with links found in with proteins of the **b** heme metabolism and **c** adipogenesis gene sets

In NEA following the differential expression analysis between patients and controls, the AGS was significantly enriched into two hallmark FGSs, adipogenesis and heme metabolism. All the AGS proteins were found at higher levels in patients (Fig. 3a). In both AGSs, the same nine proteins were involved. The set of proteins linked to adipogenesis had a substantial overlap with that from the analysis of pre-symptomatic individuals vs. controls: TNF, CSF2, CASP8, FAM81A, SELE, HTRA1, and MMP10 (Table 2). S100A12 and ORM1/ORM2 were only found in the comparison between the RA patients and controls. Both proteins were represented with two antibodies in the panel, where one of each pair was significantly differentially expressed, due to which they were included in the list.

### Gene sets identified between pre-symptomatic individuals and RA patients

**Fig. 4:**
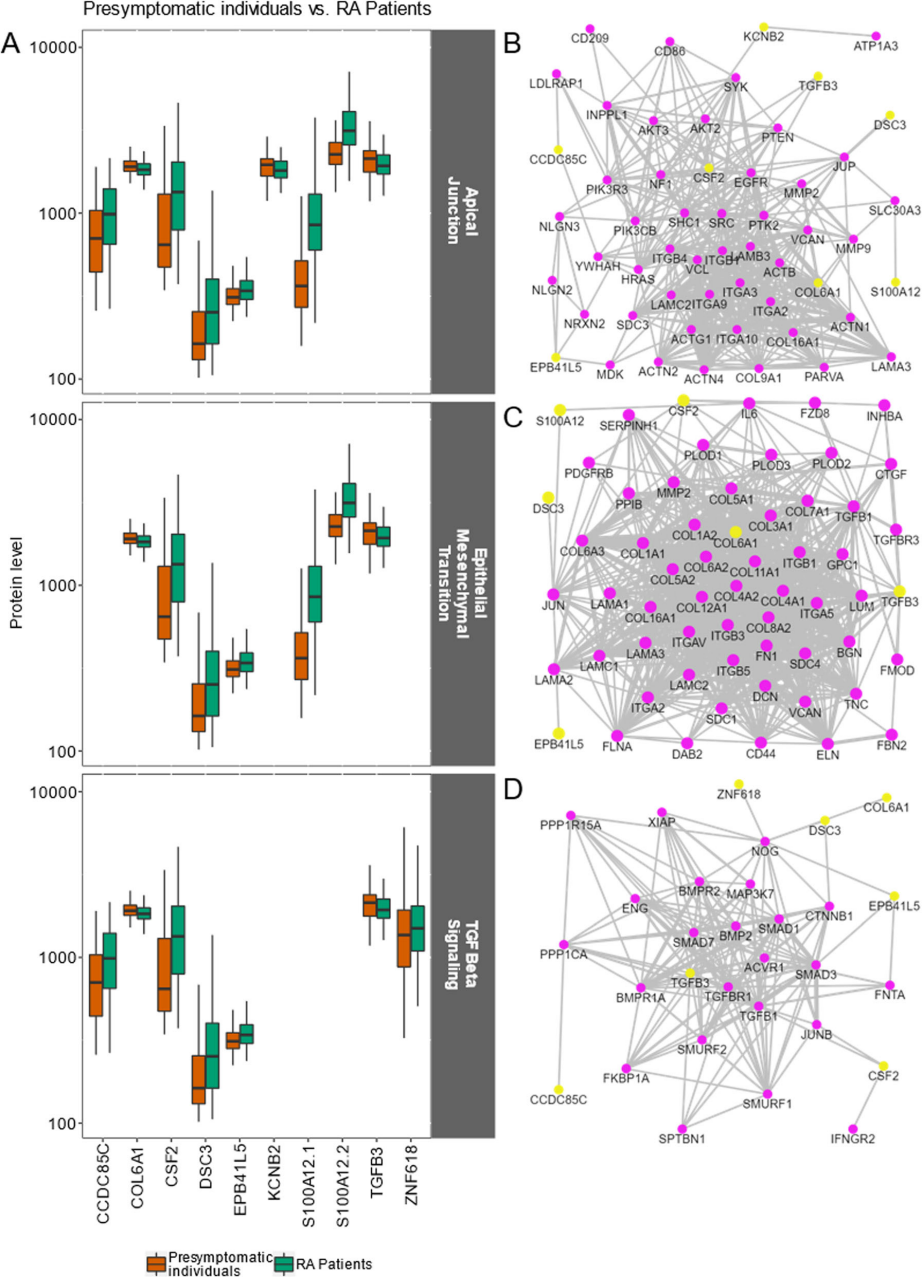
The ten AGS proteins identified in the hallmark gene sets apical junction, epithelial-mesenchymal transition, and TGF-beta signaling in pre-symptomatic individuals and RA patients. **a** Protein abundance levels (log_10_) in controls vs. pre-symptomatic individuals; the upper panel shows proteins related to apical junction, the middle panel shows proteins related to epithelial-mesenchymal transition, and the lower panel shows proteins related to the TGF-beta signaling gene set in pre-symptomatic individuals (*n* = 118, using only the sample collected closest to symptom onset) and RA patients (*n* = 74). **b**–**d** Subnetworks extracted from the global Pathway Commons network: links connecting AGS proteins (yellow) with proteins of the **b** apical junction, **c** epithelial-mesenchymal transition, and **d** TGF-beta signaling gene sets. Only S100A12.1 with the most significant results is included in graphs **b** and **c**

NEA on the AGS for pre-symptomatic individuals vs. RA patients revealed a different hallmark pattern from that of the two previously presented analyses (Table 2). The gene sets linked to the hallmarks apical junction, epithelial-mesenchymal transition, and TGF-β signaling differed between the groups (Fig. 4). In the apical junction gene set, potassium voltage-gated channel subfamily B member 2 (KCNB2), collagen type VI alpha-1 chain (COL6A1), and transforming growth factor-beta 3 (TGFB3) showed higher levels in pre-symptomatic individuals (*p* < 0.001) compared with all other proteins that were of higher levels in RA patients (*p* < 0.001–0.01) (Additional file 4: Figure S2).

## Discussion

In this study, starting with a preselected panel of 122 inflammatory and joint disease-related proteins, 107 of the proteins, detected by 153 different antibodies, remained after quality control. The network analysis could identify 6 different hallmark pathways separating the pre-symptomatic individuals from both matched controls and RA patients. Importantly, the RA patients were the same individuals as the pre-symptomatic after they had been diagnosed with RA at the early arthritis clinic. In this study, the difference in protein levels among controls, pre-symptomatic individuals, and RA patients were analyzed using both random forest modeling and linear models with subsequent analysis of NEA. To evaluate the results of the linear model analyses, we performed random forest analysis including all proteins. We observed good concordance between the 2 analyses, although random forest considered all proteins and linear models compared each protein separately.

The hallmarks separating the pre-symptomatic individuals from controls were adipogenesis and IFN-α response. In both hallmarks, all involved proteins were found in higher levels in the pre-symptomatic individuals than in the controls and 5 of them (TNF, CSF2, CASP8, SELE, HTRA1) were found in both hallmarks. Type I IFN, of which IFN-α belongs, has been shown to be altered in the development of arthritis. In a previous publication by Lubbers et al., gene expression was determined for 7 different type I IFN genes in 2 different cohorts; 3 of the analyzed genes (IFI44L, RSAD2, and EPSTI) were also included in the 97 genes related to the IFN alpha response hallmark [10]. Several of the proteins included in the hallmarks are related to inflammation and are represented within the IFN-α response innate immunity pathway. Thus, the differential expression of TNF was just modestly significant (*p* = 0.03, FDR < 0.1). However, it has been well known for involvement in the early inflammatory response and was found to be significantly increased in a previous publication where we presented the concentrations of cytokines in pre-symptomatic individuals compared with matched controls [8]. The present study revealed its central role via the statistical significance of the NEA result (Fig. 2b, c). Also, as previously presented, CSF2/GM-CSF, a pro-inflammatory cytokine that controls the production, differentiation, and function of granulocytes and monocytes, was increased in this study [8]. The blockade of this cytokine has been suggested as a therapeutic approach in RA [30]. Both cytokines are associated with the innate immune response [31]. Additionally, CASP8, an initiator of apoptosis, plays an important role in the regulation of neutrophil apoptosis and resolution of acute inflammation [32]. SELE (E-selectin), which was also found to be elevated, has been related to inflammation and TNF levels in other studies as well as to the promotion of leukocyte extravasation [33]. HTRA1 and MMP10 (the latter only within the adipogenesis hallmark) are proteins that degrade the extracellular matrix, where MMP10 is expressed and released from RA synovial fibroblasts after stimulation with adiponectin [34]. Notably, adiponectin (DIPOQ) is included in the adipogenesis hallmark as FGS (Figs. 1b and 2c; DIPOQ). Interestingly, the proteins HTRA1 and MMP10 were reported to be part of the pathogenesis process independently from the pro-inflammatory cytokines, with higher levels expressed in pre-symptomatic individuals than in controls [35]. This suggests that the degradation of the extracellular matrix is an early process occurring even before the onset of symptoms of the subsequent disease.

IL-33, a member of the IL-1 family that is also associated with innate immunity, was uniquely identified in the IFN-α pathway (Table 2). IL-33 acts as a traditional cytokine—i.e., it is upregulated in a pro-inflammatory milieu but has also been reported to act as a transcriptional regulator [36]. IL-33 stimulation primarily induces Th2 responses [36]. Thus, the observation in this study is in line with our previous report where Th2-related cytokines IL-4, IL-5, and IL-15 were shown to be upregulated in pre-symptomatic individuals [8]. Additionally, this is also supported by the observations by Hitchon et al. where early undifferentiated arthritis was shown to be associated with a Th2 response [37].

The protein PRR16 (Largen) was not included in any of the hallmark sets and has not been previously linked to RA or inflammation on the protein level. Interestingly, in RA patients from Japan, exon sequence analysis focusing on single-nucleotide variants identified the *PRR16* gene among the top 20 of 107 candidate genes for RA susceptibility [38]. The protein CCDC85C (coiled-coil domain-containing protein 85C) was not linked to any of the 2 hallmarks and has previously been identified to be increased in patients with established RA analyzed compared with osteoarthritis (OA) [12].

In our analysis, the proteins distinguishing pre-symptomatic individuals and RA patients were grouped into 3 different hallmarks—i.e., apical junction, epithelial-mesenchymal transition, and TGFβ-signaling. The protein levels of CCDC85C and CSF2/GM-CSF were different between the pre-symptomatic individuals and controls, with a higher level in the pre-symptomatic individuals. In comparison with RA patients, the levels of CCDC85C and CSF2/GM-CSF were lower in the pre-symptomatic individuals, indicating a gradual increase in these proteins as the disease develops. CCDC85C and KCNB2 have both previously been found to be altered in screening for biomarkers with significantly higher levels in RA patients compared with healthy controls and osteoarthritis (OA) patients, respectively [12]. Interestingly, in the present study, KCNB2 was found at lower levels in RA patients than in the same individual before symptom onset (pre-symptomatic individuals), suggesting a primary role of this protein in the early phase of disease development. Collagen type VI (COL6A1) is a protein present in the extracellular matrix of adipose tissue, skeletal muscle, and synovia. COL6A1, which represents 1 of the 3 alpha-chains in the heterotrimer, was also found at lower levels in RA patients than in pre-symptomatic individuals in the present study. Knowledge about COL6A1 in RA is sparse, although COL6A1, in its soluble form, has been shown to promote chondrocyte proliferation. Thus, lower detectable levels of COL6A1 could indicate impaired regeneration of the cartilage in RA patients [39]. The S100A12 protein was found to be upregulated in RA patients and has previously been shown, in the plasma together with calprotectin (S100A8/A9), to correlate with disease activity as well as with CRP in RA patients [40]. EPB41L5 (erythrocyte membrane protein band 4.1 like 5) was found in higher levels in RA patients than in pre-symptomatic individuals. It has a suggested role in the positioning of tight junctions during polarity in epithelial cells and has been identified in chronic skin disease—e.g., psoriatic vulgaris [41]. SLC11A1, another protein not included in any of the 50 hallmark gene sets, was shown by Sierra-Sanchez et al. to be increased in RA patients than in controls, a finding that is in line with our findings of increased levels in RA patients compared with pre-symptomatic individuals [13]. For the transcription factor ZNF618, interestingly, the only description we found for this protein is that the ZNF618 gene itself is located within a susceptibility region for spondylarthritis [42]. DSC3 (Desmocollin-3), a protein involved in the desmosome cell-cell junction and required for cell adhesion and desmosome formation, was found at higher levels in RA patients than in pre-symptomatic individuals in the present study. Several of the analyzed proteins—e.g., DSC3—have been suggested as candidate genes for RA susceptibility [43]. Moreover, CSF2 was found to be significantly associated with RA in a GWAS from Japan [44], and CASP8 was identified in a risk locus based on its function in immune dysregulation [45].

All the aforementioned proteins except MMP10, which only contributed to the adipogenesis gene set in pre-symptomatic individuals vs. controls, were also identified in the gene set hallmark adipogenesis and heme metabolism comparing RA patients and controls (Table 2). The same proteins that discriminated between pre-symptomatic individuals and controls, except for IL-33 and CCDC85C, were found to be significantly increased compared with that between RA patients and controls. Additionally, S100A12 and ORM1/ORM2, representing ongoing inflammation, were uniquely found in RA patients.

Our pathway analyses point toward the influence of both an innate immune response (i.e., IFN-α response) as well as the involvement of adipogenesis in initiating the events of disease development. IFN-α, which is part of the type I IFN pathway, has been linked to tissue damage, inflammation, and autoimmunity [31, 46, 47]. Our observation is in line with the reported elevated type I IFN levels in cases of arthralgia [48]. Furthermore, in untreated early cases of RA, type I IFN levels were shown to be elevated [49]. Therefore, it is plausible that, in the initiating events of the pathogenesis, increased levels of type I IFN, due to either intrinsic susceptibility or ongoing low-grade inflammation, could explain our observed difference between pre-symptomatic individuals and controls. Furthermore, inflammation, as a driver for altered lipid metabolism, has been described during infection as well as in autoimmune diseases including RA [50]. Therefore, it is of great interest that the adipogenesis pathway appears as a discriminating hallmark between pre-symptomatic individuals and controls. This observation is in line with our, and of other, previously reported altered lipid profile in pre-symptomatic individuals [51–53] as well as in patients with early arthritis [54]. In a previous study comparing RA patients, OA, and controls, lipid metabolism-related proteins differed (annexin/ANXA6 and phospholipid transfer protein/PLTP) between the groups [13]. Our group has previously shown differences in BMI and apolipoprotein alterations between pre-symptomatic individuals and matched controls [51]; however, in the present study, no impact of BMI was found in the included study groups. Thus, the involved proteins seem to participate in several processes in parallel, pinpointing the role of type I IFN responses and adipogenesis as indicators and discriminators of early pathogenesis.

Furthermore, one of the hallmarks differing between pre-symptomatic individuals and RA patients seemed to be related to cell-cell interaction and potential communication, as suggested by the apical junction hallmark [55]. This hallmark includes many adhesion molecules as well as components of the extracellular matrix, emphasizing the role of ongoing tissue remodeling during disease development. In line with this is the involvement of the epithelial-mesenchymal transition hallmark. Indeed, this pathway includes the potential contribution of inflammation to fibroblast induction and fibrosis development (reviewed in [56]. Furthermore, this is also related to TGFβ because it has been shown that TGFβ stimulates the proliferation of RA synovial fibroblasts [57]. The effect of TGFβ on synovial fibroblast proliferation has been challenged by other studies [58].

The strengths of this study include the possibility to use data from a well-defined large population-based database, with individuals who previously, and repeatedly, donated blood samples to the cohorts in the Medical Biobank before the onset of symptoms of RA. That, in combination with sampling at the time of diagnosis, provides a unique set of samples to follow the course of pre-symptomatic RA disease development.

However, we also acknowledge some limitations of this study, as the reference database MSigDB hallmark contains 50 gene sets, including approximately 7400 genes, of which not all analyzed proteins were included. The proteins we have analyzed were selected as being related to inflammation in the early phases of disease development, which could affect the results. Additionally, the number of participants included in this study was fairly low, although higher than many other similar studies using samples from pre-symptomatic individuals.

## Conclusions

In summary, using NEA, we have found new proteins and their network partners, in particular, those involved in tissue remodeling, as well as confirmed previously reported proteins such as TNF. Our study provides an in-depth analysis of potential involved candidate proteins in the development of the complex disease rheumatoid arthritis.

## Supplementary information

**Additional file 1: Table S1.** Demographic data for the 118 pre-symptomatic individuals, 79 RA patients and 74 control subjects.

**Additional file 2: Table S2.** List of the included 153 antibodies detecting 107 different proteins, their corresponding gene name, gene descriptions, ENSG id, and *p*-values from the multifactorial linear regression for the three two-group comparisons.

**Additional file 3: Figure S1.** Multidimensional scaling using random forest modeling (summarizing all factors—i.e., proteins), demonstrating the clustering of control subjects, pre-symptomatic individuals (pre-patients), and patients. Pre-symptomatic individuals were defined as individuals in whom symptoms of rheumatoid arthritis (RA) had not yet occurred; patients were defined as the same individuals after the onset of RA. The 2 axes represent the dominant clustering directions between the groups.

**Additional file 4: Figure S2.** Expression levels (log base 2) of the 19 distinct proteins identified via 21 antibodies from the lists of the ten most significant proteins in the three two-level linear models contrasts. The graph display the individuals’ values for controls, pre-symptomatic individuals and patients (depicted as 0, 1 or 2) across the time of sampling, expressed in years before symptom onset. Because controls do not have any date of symptom onset, they were assigned normally distributed random time values, always lower than the earliest pre-symptomatic sample. *P*-values estimate the significance of differences from the respective 2-level contrasts in multifactorial linear models models. FCs are log base 2-fold change values of protein expression, where positive numbers correspond to higher expression in the rightmost group.

## Data Availability

The datasets generated and/or analyzed during the current study are not publicly available due to the risk of identifying study participants using the anonymized data and, also, as the studies on individuals before disease onset will be included in another study.

## Abbreviations

AGS: Altered gene set
anti-CCP: Anti-cyclic citrullinated peptide antibodies
AUC: Area under the curve
CASP8: Caspase 8
CCDC85C: Coiled-coil domain containing 85C
COL6A1: Collagen type VI alpha 1 chain
CSF2: Colony-stimulating factor 2
DSC3: Desmocollin 3
EPB41L5: Erythrocyte membrane protein band 4.1 like 5
FAM81A: Family with sequence similarity 81 member A
FGS: Functional gene set
HTRA1: HtrA serine peptidase 1
IL33: Interleukin 33
KCNB2: Potassium voltage-gated channel subfamily B member 2
MMP10: Matrix metallopeptidase 10
NEA: Network enrichment analysis
ORM1: Orosomucoid 1
ORM2: Orosomucoid 2
PRR16: Proline rich 16
RA: Rheumatoid arthritis
S100A12: S100 calcium-binding protein A12
SELE: Selectin E
SLC11A1: Solute carrier family 11 member 1
TGFB3: Transforming growth factor beta 3
TNF: Tumor necrosis factor
ZNF618: Zinc finger protein 618
