# Supplementary material for: Protein profiling and network enrichment analysis in individuals before and after the onset of rheumatoid arthritis
Source: Arthritis Res Ther. 2019 Dec 16;21:288. doi: 10.1186/s13075-019-2066-9 (PMC6915963; doi:10.1186/s13075-019-2066-9)
Supplement: Supplementary file 1 — Additional file 1: Table S1. Demographic data for the 118 pre-symptomatic individuals, 79 RA patients and 74 control subjects. [file 13075_2019_2066_MOESM1_ESM.docx]

|  | Controls  n = 74 | Presymptomatic individuals ^d^  n = 118 | Patients  n = 79 |
| --- | --- | --- | --- |
| Sex, females n (%) | 60 (81.1) | 102 (86.4) | 65 (82.3) |
| Age at sampling (years), median (IQR) | 59.8 (9.9) | 58.8 (8.8) | 61.9 (13.4)^b^ |
| Ever smoker, yes n (%) | 30 (40.5) | 69 (58.5)^a^ | 50 (63.3)^b^ |
| BMI (kg/m^2^), mean (SD) | 25.9 (3.1) | 26.6 (4.7) | 26.8 (5.0) |
| HLA-SE^c^, positive n (%) | - | 79 (67.5) | 54 (68.4) |
| Rheumatoid Factor positive, n (%) | - | 51 (45.1)^a^ | 68 (86.1)^b^ |
| Anti-CCP2 positive, n (%) | 2 (2.7) | 65 (55.1)^a^ | 66 (83.5)^b^ |
| DAS28, mean (SD) | - | - | 4.9 (1.4) |
| ESR (mm/h), mean (SD) | - | - | 32.9 (24) |
| CRP (mg/mL), mean (SD) | - | - | 24.2 (28.0) |
| Duration of symptoms before the diagnosis of RA (months), median (IQR) | - | 6.42 (5.2) | - |
| Time between sampling and onset of symptoms (years), median (IQR) | - | 3.1 (3.6) | - |
| ^a^p<0.05 presymptomatic individuals compared with controls.  ^b^p<0.05 RA patients compared with controls.  ^c^HLA-shared epitope = HLA-B1*0101/0401/0404/0405/0408.  ^d^Calculated on the sample closest to symptom onset; median: 3.1 years. | | | |

**Table S1.**
