# Supplementary material for: Protein profiling and network enrichment analysis in individuals before and after the onset of rheumatoid arthritis
Source: Arthritis Res Ther. 2019 Dec 16;21:288. doi: 10.1186/s13075-019-2066-9 (PMC6915963; doi:10.1186/s13075-019-2066-9)
Supplement: Supplementary file 2 — Additional file 2: Table S2. List of the included 153 antibodies detecting 107 different proteins, their corresponding gene name, gene descriptions, ENSG id, and p-values from the multifactorial linear regression for the three two-group comparisons. [file 13075_2019_2066_MOESM2_ESM.docx]

**Table S2.**

| HPA-id | Gene name | Gene Description | ENSG id | p-value,  pre-symptomatic individuals vs. control | p-value,  RA-patient vs. control | p-value,  RA vs. pre-symptomatic individuals |
| --- | --- | --- | --- | --- | --- | --- |
| HPA064436 | AGER | advanced glycosylation end-product specific receptor | ENSG00000204305 | 1,46E-01 | 5,34E-03 | 2,18E-01 |
| HPA032147 | ANO1 | anoctamin 1 | ENSG00000131620 | 1,31E-02 | 4,37E-10 | 2,74E-01 |
| HPA057356 | ANO1 | anoctamin 1 | ENSG00000131620 | 7,31E-04 | 7,60E-14 | 1,49E-01 |
| HPA059335 | ANOS1 | anosmin 1 | ENSG00000011201 | 2,22E-03 | 4,07E-03 | 7,06E-01 |
| HPA068656 | ANOS1 | anosmin 1 | ENSG00000011201 | 1,55E-03 | 9,90E-12 | 1,56E-01 |
| HPA046715 | APOA1 | apolipoprotein A1 | ENSG00000118137 | 1,28E-01 | 5,40E-04 | 3,78E-01 |
| HPA046415 | BGLAP | bone gamma-carboxyglutamate protein | ENSG00000242252 | 8,47E-01 | 5,32E-04 | 1,85E-01 |
| HPA063980 | CHST14 | carbohydrate sulfotransferase 14 | ENSG00000169105 | 6,11E-01 | 9,23E-01 | 4,60E-01 |
| HPA071601 | CHST14 | carbohydrate sulfotransferase 14 | ENSG00000169105 | 6,76E-05 | 2,39E-13 | 1,45E-02 |
| HPA003056 | CASP1 | caspase 1 | ENSG00000137752 | 1,35E-02 | 2,09E-10 | 6,56E-01 |
| HPA001302 | CASP8 | caspase 8 | ENSG00000064012 | 3,72E-06 | 2,35E-23 | 3,29E-02 |
| HPA005688 | CASP8 | caspase 8 | ENSG00000064012 | 6,40E-01 | 1,88E-02 | 6,08E-01 |
| HPA042071 | CARD8 | caspase recruitment domain family member 8 | ENSG00000105483 | 2,14E-03 | 8,04E-17 | 7,90E-02 |
| HPA043513 | CARD8 | caspase recruitment domain family member 8 | ENSG00000105483 | 2,85E-02 | 2,15E-03 | 3,41E-01 |
| HPA011652 | CCL11 | C-C motif chemokine ligand 11 | ENSG00000172156 | 7,88E-01 | 2,77E-02 | 4,76E-02 |
| HPA019163 | CCL2 | C-C motif chemokine ligand 2 | ENSG00000108691 | 7,57E-04 | 3,84E-11 | 2,23E-01 |
| HPA063758 | CCL23 | C-C motif chemokine ligand 23 | ENSG00000274736 | 9,42E-01 | 5,56E-02 | 2,43E-02 |
| HPA067937 | CEBPA | CCAAT/enhancer binding protein alpha | ENSG00000245848 | 3,58E-01 | 2,83E-02 | 4,51E-01 |
| HPA060839 | CD5 | CD5 molecule | ENSG00000110448 | 3,97E-01 | 3,36E-02 | 9,98E-01 |
| HPA044335 | CENPF | centromere protein F | ENSG00000117724 | 5,10E-02 | 1,94E-07 | 1,48E-01 |
| HPA063233 | CENPF | centromere protein F | ENSG00000117724 | 9,54E-05 | 1,62E-15 | 5,02E-02 |
| HPA064308 | CENPF | centromere protein F | ENSG00000117724 | 1,55E-01 | 1,42E-04 | 7,59E-01 |
| HPA010575 | CHIT1 | chitinase 1 | ENSG00000133063 | 3,93E-03 | 3,16E-07 | 3,23E-01 |
| HPA027499 | C1orf106 | chromosome 1 open reading frame 106 | ENSG00000163362 | 1,48E-02 | 2,48E-04 | 8,15E-01 |
| HPA027511 | C1orf106 | chromosome 1 open reading frame 106 | ENSG00000163362 | 6,68E-01 | 7,89E-02 | 6,11E-02 |
| HPA058346 | CCDC85C | coiled-coil domain containing 85C | ENSG00000205476 | 2,91E-06 | 3,04E-19 | 6,28E-03 |
| HPA019142 | COL6A1 | collagen type VI alpha 1 chain | ENSG00000142156 | 4,28E-01 | 1,11E-01 | 2,55E-03 |
| HPA022244 | CSF1 | colony stimulating factor 1 | ENSG00000184371 | 1,80E-02 | 2,49E-07 | 2,10E-01 |
| HPA039288 | CSF2 | colony stimulating factor 2 | ENSG00000164400 | 2,05E-06 | 3,33E-24 | 6,73E-03 |
| HPA048058 | CSF2 | colony stimulating factor 2 | ENSG00000164400 | 9,46E-05 | 8,76E-17 | 1,05E-02 |
| HPA040361 | CX3CL1 | C-X3-C motif chemokine ligand 1 | ENSG00000006210 | 4,60E-02 | 5,56E-07 | 3,82E-01 |
| HPA056729 | CX3CL1 | C-X3-C motif chemokine ligand 1 | ENSG00000006210 | 8,44E-02 | 3,90E-02 | 1,20E-01 |
| HPA054954 | CXCL10 | C-X-C motif chemokine ligand 10 | ENSG00000169245 | 2,85E-01 | 1,30E-03 | 7,55E-02 |
| HPA057179 | CXCL8 | C-X-C motif chemokine ligand 8 | ENSG00000169429 | 8,72E-01 | 4,13E-01 | 8,59E-01 |
| HPA003603 | CXCL9 | C-X-C motif chemokine ligand 9 | ENSG00000138755 | 4,85E-02 | 1,17E-03 | 1,44E-01 |
| HPA070860 | CXCL9 | C-X-C motif chemokine ligand 9 | ENSG00000138755 | 9,31E-02 | 1,55E-04 | 9,53E-01 |
| HPA047688 | CCNI2 | cyclin I family member 2 | ENSG00000205089 | 9,63E-04 | 7,73E-12 | 2,55E-01 |
| HPA056288 | CCNI2 | cyclin I family member 2 | ENSG00000205089 | 1,02E-01 | 9,01E-02 | 7,78E-01 |
| HPA049265 | DSC3 | desmocollin 3 | ENSG00000134762 | 2,09E-04 | 8,57E-16 | 6,90E-03 |
| HPA066836 | ELANE | elastase, neutrophil expressed | ENSG00000197561 | 9,28E-02 | 1,91E-09 | 9,82E-01 |
| HPA037563 | EPB41L5 | erythrocyte membrane protein band 4.1 like 5 | ENSG00000115109 | 5,33E-02 | 5,11E-07 | 4,11E-01 |
| HPA037564 | EPB41L5 | erythrocyte membrane protein band 4.1 like 5 | ENSG00000115109 | 4,85E-01 | 4,42E-05 | 1,97E-03 |
| HPA028484 | EXOSC10 | exosome component 10 | ENSG00000171824 | 2,24E-05 | 5,68E-19 | 7,42E-01 |
| HPA027241 | ECM1 | extracellular matrix protein 1 | ENSG00000143369 | 9,16E-04 | 1,52E-17 | 3,14E-02 |
| HPA048814 | FAM81A | family with sequence similarity 81 member A | ENSG00000157470 | 6,52E-01 | 5,23E-01 | 6,62E-02 |
| HPA065797 | FAM81A | family with sequence similarity 81 member A | ENSG00000157470 | 5,77E-06 | 6,74E-22 | 1,60E-02 |
| HPA057930 | FETUB | fetuin B | ENSG00000090512 | 7,73E-03 | 1,06E-10 | 1,57E-02 |
| HPA051370 | FGA | fibrinogen alpha chain | ENSG00000171560 | 4,07E-03 | 3,56E-11 | 3,93E-02 |
| HPA064755 | FGA | fibrinogen alpha chain | ENSG00000171560 | 1,00E-01 | 3,41E-05 | 5,54E-02 |
| HPA053564 | FILIP1 | filamin A interacting protein 1 | ENSG00000118407 | 8,71E-04 | 4,27E-11 | 1,55E-01 |
| HPA060985 | GDF11 | growth differentiation factor 11 | ENSG00000135414 | 4,82E-02 | 1,04E-04 | 4,17E-01 |
| HPA069609 | GDF11 | growth differentiation factor 11 | ENSG00000135414 | 2,38E-01 | 3,89E-03 | 3,74E-01 |
| HPA002184 | HTRA1 | HtrA serine peptidase 1 | ENSG00000166033 | 1,39E-05 | 2,05E-20 | 1,55E-02 |
| HPA023072 | IDO1 | indoleamine 2,3-dioxygenase 1 | ENSG00000131203 | 6,91E-01 | 3,86E-01 | 2,70E-02 |
| HPA027772 | IDO1 | indoleamine 2,3-dioxygenase 1 | ENSG00000131203 | 3,34E-03 | 8,78E-13 | 7,59E-01 |
| HPA049525 | IFNG | interferon gamma | ENSG00000111537 | 1,01E-02 | 3,93E-11 | 1,67E-01 |
| HPA053530 | IFNG | interferon gamma | ENSG00000111537 | 2,17E-05 | 1,96E-15 | 8,40E-02 |
| HPA001410 | IL1B | interleukin 1 beta | ENSG00000125538 | 8,21E-03 | 3,30E-11 | 6,77E-01 |
| HPA075019 | IL1B | interleukin 1 beta | ENSG00000125538 | 2,11E-02 | 1,39E-09 | 1,18E-01 |
| HPA051182 | IL10 | interleukin 10 | ENSG00000136634 | 2,41E-01 | 1,08E-05 | 7,77E-01 |
| HPA063270 | IL10 | interleukin 10 | ENSG00000136634 | 1,10E-01 | 8,23E-03 | 1,66E-01 |
| HPA065647 | IL10RA | interleukin 10 receptor subunit alpha | ENSG00000110324 | 6,49E-01 | 9,70E-02 | 7,41E-02 |
| HPA069086 | IL10RA | interleukin 10 receptor subunit alpha | ENSG00000110324 | 1,92E-01 | 1,73E-04 | 3,94E-02 |
| HPA041100 | IL12B | interleukin 12B | ENSG00000113302 | 1,91E-03 | 6,61E-16 | 2,14E-02 |
| HPA042421 | IL13 | interleukin 13 | ENSG00000169194 | 9,11E-02 | 2,44E-09 | 1,81E-01 |
| HPA072425 | IL17RB | interleukin 17 receptor B | ENSG00000056736 | 4,84E-02 | 4,11E-02 | 5,22E-01 |
| HPA052258 | IL17A | interleukin 17A | ENSG00000112115 | 3,08E-01 | 6,27E-01 | 9,26E-02 |
| HPA003980 | IL18 | interleukin 18 | ENSG00000150782 | 5,29E-01 | 4,90E-01 | 1,54E-01 |
| HPA054622 | IL2RA | interleukin 2 receptor subunit alpha | ENSG00000134460 | 4,50E-02 | 7,76E-06 | 6,55E-01 |
| HPA031387 | IL20 | interleukin 20 | ENSG00000162891 | 1,22E-01 | 5,01E-04 | 2,88E-01 |
| HPA067147 | IL20 | interleukin 20 | ENSG00000162891 | 6,20E-02 | 7,10E-06 | 5,71E-01 |
| HPA038303 | IL21 | interleukin 21 | ENSG00000138684 | 5,13E-01 | 6,75E-02 | 2,50E-01 |
| HPA056628 | IL21 | interleukin 21 | ENSG00000138684 | 3,97E-01 | 8,38E-02 | 8,80E-01 |
| HPA042399 | IL22RA1 | interleukin 22 receptor subunit alpha 1 | ENSG00000142677 | 7,26E-02 | 8,62E-01 | 4,24E-01 |
| HPA073260 | IL24 | interleukin 24 | ENSG00000162892 | 9,53E-02 | 7,69E-05 | 7,82E-01 |
| HPA069269 | IL26 | interleukin 26 | ENSG00000111536 | 4,31E-04 | 1,85E-15 | 1,20E-02 |
| HPA052386 | IL33 | interleukin 33 | ENSG00000137033 | 5,45E-06 | 3,62E-16 | 2,06E-02 |
| HPA042270 | IL4 | interleukin 4 | ENSG00000113520 | 6,53E-01 | 2,19E-02 | 1,89E-01 |
| HPA063382 | IL4 | interleukin 4 | ENSG00000113520 | 5,62E-03 | 1,13E-15 | 8,44E-03 |
| HPA070010 | IL4 | interleukin 4 | ENSG00000113520 | 2,06E-01 | 4,84E-02 | 3,21E-01 |
| HPA065029 | IL5 | interleukin 5 | ENSG00000113525 | 5,94E-01 | 1,10E-01 | 3,12E-01 |
| HPA060030 | IL6 | interleukin 6 | ENSG00000136244 | 7,23E-01 | 8,37E-02 | 3,08E-01 |
| HPA042855 | IL7 | interleukin 7 | ENSG00000104432 | 3,36E-03 | 7,81E-06 | 2,44E-02 |
| HPA035516 | KIF15 | kinesin family member 15 | ENSG00000163808 | 9,47E-02 | 8,23E-02 | 8,42E-01 |
| HPA061469 | KIF15 | kinesin family member 15 | ENSG00000163808 | 1,26E-03 | 5,90E-14 | 2,06E-02 |
| HPA070395 | KITLG | KIT ligand | ENSG00000049130 | 5,54E-01 | 1,03E-01 | 4,21E-02 |
| HPA057322 | LEP | leptin | ENSG00000174697 | 2,10E-02 | 7,08E-09 | 2,48E-02 |
| HPA068565 | LEP | leptin | ENSG00000174697 | 4,87E-03 | 4,04E-09 | 2,11E-01 |
| HPA031456 | MMP1 | matrix metallopeptidase 1 | ENSG00000196611 | 6,68E-02 | 7,53E-07 | 9,20E-01 |
| HPA052343 | MMP10 | matrix metallopeptidase 10 | ENSG00000166670 | 2,29E-05 | 1,56E-21 | 2,91E-02 |
| HPA053433 | MMP10 | matrix metallopeptidase 10 | ENSG00000166670 | 2,16E-05 | 3,53E-19 | 2,60E-02 |
| HPA068962 | MMP13 | matrix metallopeptidase 13 | ENSG00000137745 | 5,12E-02 | 3,05E-05 | 5,15E-01 |
| HPA051358 | MMP7 | matrix metallopeptidase 7 | ENSG00000137673 | 8,03E-02 | 2,75E-01 | 1,42E-01 |
| HPA063662 | MMP7 | matrix metallopeptidase 7 | ENSG00000137673 | 4,06E-02 | 1,68E-05 | 4,12E-01 |
| HPA063909 | MMP9 | matrix metallopeptidase 9 | ENSG00000100985 | 3,75E-02 | 4,09E-05 | 1,75E-01 |
| HPA041093 | MEX3B | mex-3 RNA binding family member B | ENSG00000183496 | 1,58E-01 | 5,59E-06 | 2,18E-02 |
| HPA039062 | MAP6 | microtubule associated protein 6 | ENSG00000171533 | 3,02E-01 | 7,69E-02 | 1,19E-01 |
| HPA067854 | MAP2K3,  MAP2K6 | mitogen-activated protein kinase kinase 3,mitogen-activated protein kinase kinase 6 | ENSG00000034152,  ENSG00000108984 | 6,43E-04 | 1,76E-11 | 8,24E-01 |
| HPA031134 | MAP2K6 | mitogen-activated protein kinase kinase 6 | ENSG00000108984 | 3,88E-02 | 9,27E-05 | 7,39E-01 |
| HPA021021 | MURC | muscle related coiled-coil protein | ENSG00000170681 | 1,19E-02 | 3,14E-09 | 7,41E-01 |
| HPA045280 | NAPSA | napsin A aspartic peptidase | ENSG00000131400 | 1,40E-02 | 8,73E-09 | 2,20E-01 |
| HPA063135 | NGF | nerve growth factor | ENSG00000134259 | 9,11E-02 | 1,57E-02 | 8,32E-01 |
| HPA047725 | ORM2,  ORM1 | orosomucoid 2,orosomucoid 1 | ENSG00000228278,  ENSG00000229314 | 4,14E-05 | 5,80E-20 | 1,15E-02 |
| HPA051056 | PIK3C2G | phosphatidylinositol-4-phosphate 3-kinase catalytic subunit type 2 gamma | ENSG00000139144 | 1,68E-03 | 8,79E-14 | 1,83E-01 |
| HPA066051 | PIK3C2G | phosphatidylinositol-4-phosphate 3-kinase catalytic subunit type 2 gamma | ENSG00000139144 | 6,44E-03 | 5,45E-10 | 1,75E-02 |
| HPA047815 | PLPP1 | phospholipid phosphatase 1 | ENSG00000067113 | 4,21E-01 | 3,71E-01 | 2,82E-02 |
| HPA018161 | KCNB2 | potassium voltage-gated channel subfamily B member 2 | ENSG00000182674 | 1,30E-01 | 1,10E-02 | 8,26E-02 |
| HPA070273 | KCNB2 | potassium voltage-gated channel subfamily B member 2 | ENSG00000182674 | 4,19E-01 | 4,79E-02 | 2,92E-04 |
| HPA049254 | PRR16 | proline rich 16 | ENSG00000184838 | 2,68E-07 | 9,82E-26 | 1,77E-02 |
| HPA059390 | PRR16 | proline rich 16 | ENSG00000184838 | 6,02E-04 | 4,16E-15 | 4,55E-02 |
| HPA054496 | PYCARD | PYD and CARD domain containing | ENSG00000103490 | 7,31E-02 | 2,05E-02 | 8,03E-01 |
| HPA041725 | RBBP6 | RB binding protein 6, ubiquitin ligase | ENSG00000122257 | 1,64E-02 | 3,65E-08 | 4,13E-01 |
| HPA069236 | RBBP6 | RB binding protein 6, ubiquitin ligase | ENSG00000122257 | 2,46E-04 | 2,49E-16 | 4,18E-02 |
| HPA002881 | S100A12 | S100 calcium binding protein A12 | ENSG00000163221 | 4,88E-02 | 4,91E-14 | 4,32E-03 |
| HPA003620 | S100A12 | S100 calcium binding protein A12 | ENSG00000163221 | 6,06E-04 | 1,06E-24 | 7,41E-04 |
| HPA064231 | SOST | sclerostin | ENSG00000167941 | 4,35E-02 | 5,09E-03 | 4,34E-01 |
| HPA068849 | SOST | sclerostin | ENSG00000167941 | 9,68E-01 | 1,01E-02 | 2,56E-02 |
| HPA040196 | SEC24C | SEC24 homolog C, COPII coat complex component | ENSG00000176986 | 6,55E-01 | 8,84E-01 | 8,67E-01 |
| HPA027541 | SPP1 | secreted phosphoprotein 1 | ENSG00000118785 | 1,34E-01 | 1,30E-05 | 2,30E-01 |
| HPA006225 | SELE | selectin E | ENSG00000007908 | 6,33E-03 | 4,95E-08 | 9,47E-01 |
| HPA027227 | SELE | selectin E | ENSG00000007908 | 8,44E-06 | 2,30E-20 | 8,67E-02 |
| HPA051251 | SGK1 | serum/glucocorticoid regulated kinase 1 | ENSG00000118515 | 2,03E-03 | 5,62E-09 | 5,56E-01 |
| HPA029590 | SLC11A1 | solute carrier family 11 member 1 | ENSG00000018280 | 1,45E-01 | 1,02E-02 | 1,77E-01 |
| HPA068540 | SLC11A1 | solute carrier family 11 member 1 | ENSG00000018280 | 3,23E-02 | 1,74E-08 | 8,33E-03 |
| HPA030489 | SLC39A2 | solute carrier family 39 member 2 | ENSG00000165794 | 3,63E-01 | 1,02E-02 | 7,12E-02 |
| HPA073231 | SLC39A2 | solute carrier family 39 member 2 | ENSG00000165794 | 3,91E-01 | 2,59E-04 | 7,79E-02 |
| HPA066376 | SFN,  YWHAE,  YWHAQ,  YWHAH,  YWHAB,  YWHAG,  YWHAZ | stratifin, tyrosine 3-monooxygenase/tryptophan 5-monooxygenase activation protein epsilon/theta/eta/beta/gamma/zeta | ENSG00000175793, ENSG00000108953,  ENSG00000134308, ENSG00000128245,  ENSG00000166913, ENSG00000170027,  ENSG00000164924 | 5,28E-01 | 8,17E-01 | 1,35E-01 |
| HPA056350 | TSLP | thymic stromal lymphopoietin | ENSG00000145777 | 1,42E-01 | 1,70E-05 | 1,45E-01 |
| HPA012314 | TNFRSF11A | TNF receptor superfamily member 11a | ENSG00000141655 | 9,42E-05 | 1,81E-14 | 3,98E-02 |
| HPA027728 | TNFRSF11A | TNF receptor superfamily member 11a | ENSG00000141655 | 5,82E-01 | 6,90E-03 | 4,07E-01 |
| HPA058613 | TNFRSF11B | TNF receptor superfamily member 11b | ENSG00000164761 | 5,81E-01 | 2,01E-01 | 1,70E-01 |
| HPA051188 | TLR2 | toll like receptor 2 | ENSG00000137462 | 1,20E-03 | 3,08E-09 | 1,96E-02 |
| HPA060231 | TLR2 | toll like receptor 2 | ENSG00000137462 | 9,34E-04 | 1,81E-06 | 6,20E-02 |
| HPA055602 | TGFA | transforming growth factor alpha | ENSG00000163235 | 4,99E-02 | 1,45E-05 | 5,93E-01 |
| HPA063582 | TGFB3 | transforming growth factor beta 3 | ENSG00000119699 | 5,81E-01 | 8,21E-02 | 4,43E-03 |
| HPA044863 | TSPYL4 | TSPY like 4 | ENSG00000187189 | 2,88E-02 | 4,23E-11 | 2,76E-02 |
| HPA050631 | TNF | tumor necrosis factor | ENSG00000232810 | 3,11E-02 | 1,74E-04 | 4,21E-01 |
| HPA064998 | TNF | tumor necrosis factor | ENSG00000232810 | 1,94E-07 | 5,52E-26 | 1,65E-02 |
| HPA054938 | TNFSF10 | tumor necrosis factor superfamily member 10 | ENSG00000121858 | 7,39E-03 | 9,84E-18 | 1,54E-02 |
| HPA068318 | TNFSF10 | tumor necrosis factor superfamily member 10 | ENSG00000121858 | 2,53E-02 | 7,00E-07 | 8,08E-02 |
| HPA065635 | TNFSF13 | tumor necrosis factor superfamily member 13 | ENSG00000161955 | 1,74E-01 | 3,69E-01 | 2,65E-01 |
| HPA066440 | TNFSF13B | tumor necrosis factor superfamily member 13b | ENSG00000102524 | 3,55E-01 | 6,35E-01 | 1,85E-01 |
| HPA071388 | TNFSF13B | tumor necrosis factor superfamily member 13b | ENSG00000102524 | 1,55E-04 | 3,03E-12 | 2,56E-02 |
| HPA074859 | UBE2O | ubiquitin conjugating enzyme E2 O | ENSG00000175931 | 4,13E-01 | 2,73E-01 | 3,61E-01 |
| HPA071077 | UCMA | upper zone of growth plate and cartilage matrix associated | ENSG00000165623 | 5,20E-01 | 4,24E-01 | 4,37E-02 |
| HPA039690 | WHAMM | WAS protein homolog associated with actin, golgi membranes and microtubules | ENSG00000156232 | 8,34E-01 | 9,53E-01 | 1,59E-01 |
| HPA040231 | WHAMM | WAS protein homolog associated with actin, golgi membranes and microtubules | ENSG00000156232 | 1,79E-01 | 2,55E-03 | 2,03E-01 |
| HPA034796 | VCAM1 | vascular cell adhesion molecule 1 | ENSG00000162692 | 6,17E-02 | 6,08E-09 | 8,54E-02 |
| HPA069867 | VCAM1 | vascular cell adhesion molecule 1 | ENSG00000162692 | 9,42E-01 | 3,76E-01 | 2,39E-01 |
| HPA053984 | VEGFA | vascular endothelial growth factor A | ENSG00000112715 | 3,93E-01 | 5,70E-02 | 4,55E-01 |
| HPA063302 | VEGFA | vascular endothelial growth factor A | ENSG00000112715 | 5,90E-03 | 6,40E-07 | 5,97E-01 |
| HPA063777 | VCL | vinculin | ENSG00000035403 | 5,96E-05 | 1,48E-16 | 2,96E-02 |
| HPA061732 | ZNF618 | zinc finger protein 618 | ENSG00000157657 | 7,85E-02 | 1,40E-04 | 3,82E-03 |
| Levels of significance after adjustment for multiple testing (by Benjamini-Hochberg):  Pre-symptomatic individuals vs. Control: P-value cut-off for FDR=0.001 is 0.000155 (19 proteins)  Pre-symptomatic individuals vs. Control: P-value cut-off for FDR=0.01 is 0.00222 (40 proteins)  Pre-symptomatic individuals vs. Control: P-value cut-off for FDR=0.1 is 0.0533 (80 proteins)  RA vs. Control: P-value cut-off for FDR=0.001 is 0.00054 (96 proteins)  RA vs. Control: P-value cut-off for FDR=0.01 is 0.0069 (104 proteins)  RA vs. Control: P-value cut-off for FDR=0.1 is 0.0838 (130 proteins)  RA vs. Pre-symptomatic individuals: P-value cut-off for FDR=0.1 is 0.00382 (7 proteins) | | | | | | |
