# Supplementary material for: Protein profiling and network enrichment analysis in individuals before and after the onset of rheumatoid arthritis
Source: Arthritis Res Ther. 2019 Dec 16;21:288. doi: 10.1186/s13075-019-2066-9 (PMC6915963; doi:10.1186/s13075-019-2066-9)

**Figure S1**

1.
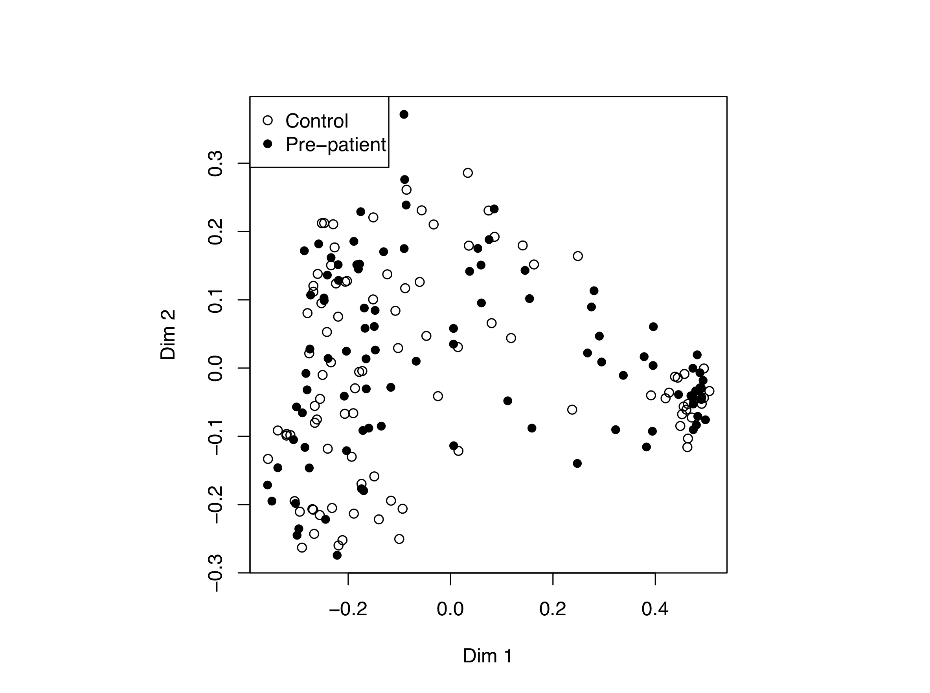
Pre-symptomatic individuals and controls
2. RA patients and Controls


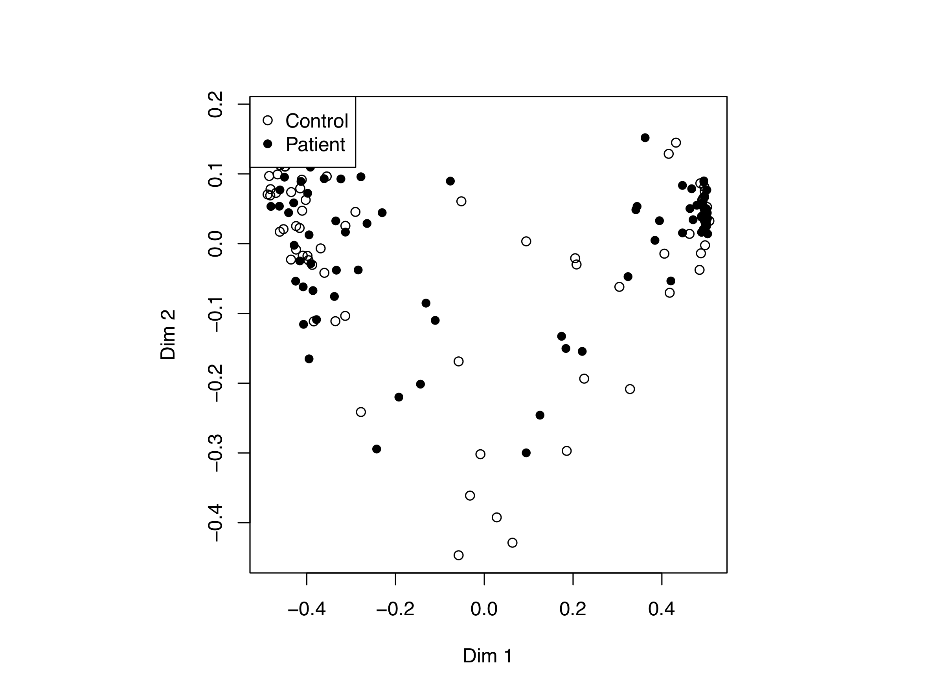


1. Pre-symptomatic individuals and RA patients.


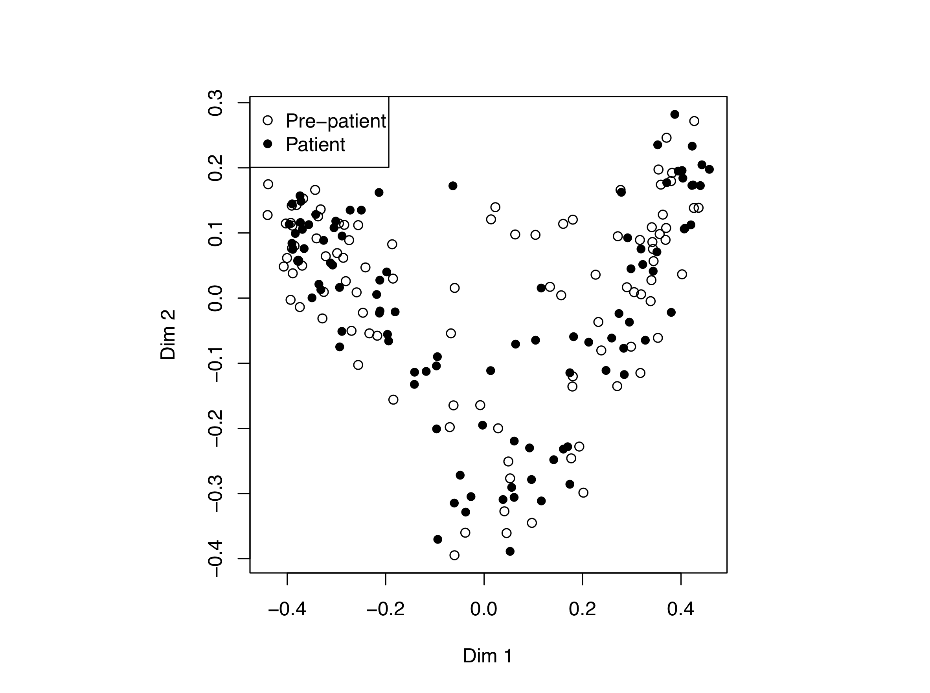

Supplement: Supplementary file 3 — Additional file 3: Figure S1. Multidimensional scaling using random forest modeling (summarizing all factors—i.e., proteins), demonstrating the clustering of control subjects, pre-symptomatic individuals (pre-patients), and patients. Pre-symptomatic individuals were defined as individuals in whom symptoms of rheumatoid arthritis (RA) had not yet occurred; patients were defined as the same individuals after the onset of RA. The 2 axes represent the dominant clustering directions between the groups. [file 13075_2019_2066_MOESM3_ESM.docx]
