# Supplementary material for: Protein profiling and network enrichment analysis in individuals before and after the onset of rheumatoid arthritis
Source: Arthritis Res Ther. 2019 Dec 16;21:288. doi: 10.1186/s13075-019-2066-9 (PMC6915963; doi:10.1186/s13075-019-2066-9)
Supplement: Supplementary file 4 — Additional file 4: Figure S2. Expression levels (log base 2) of the 19 distinct proteins identified via 21 antibodies from the lists of the ten most significant proteins in the three two-level linear models contrasts. The graph display the individuals’ values for controls, pre-symptomatic individuals and patients (depicted as 0, 1 or 2) across the time of sampling, expressed in years before symptom onset. Because controls do not have any date of symptom onset, they were assigned normally distributed random time values, always lower than the earliest pre-symptomatic sample. P-values estimate the significance of differences from the respective 2-level contrasts in multifactorial linear models models. FCs are log base 2-fold change values of protein expression, where positive numbers correspond to higher expression in the rightmost group. [file 13075_2019_2066_MOESM4_ESM.pdf]

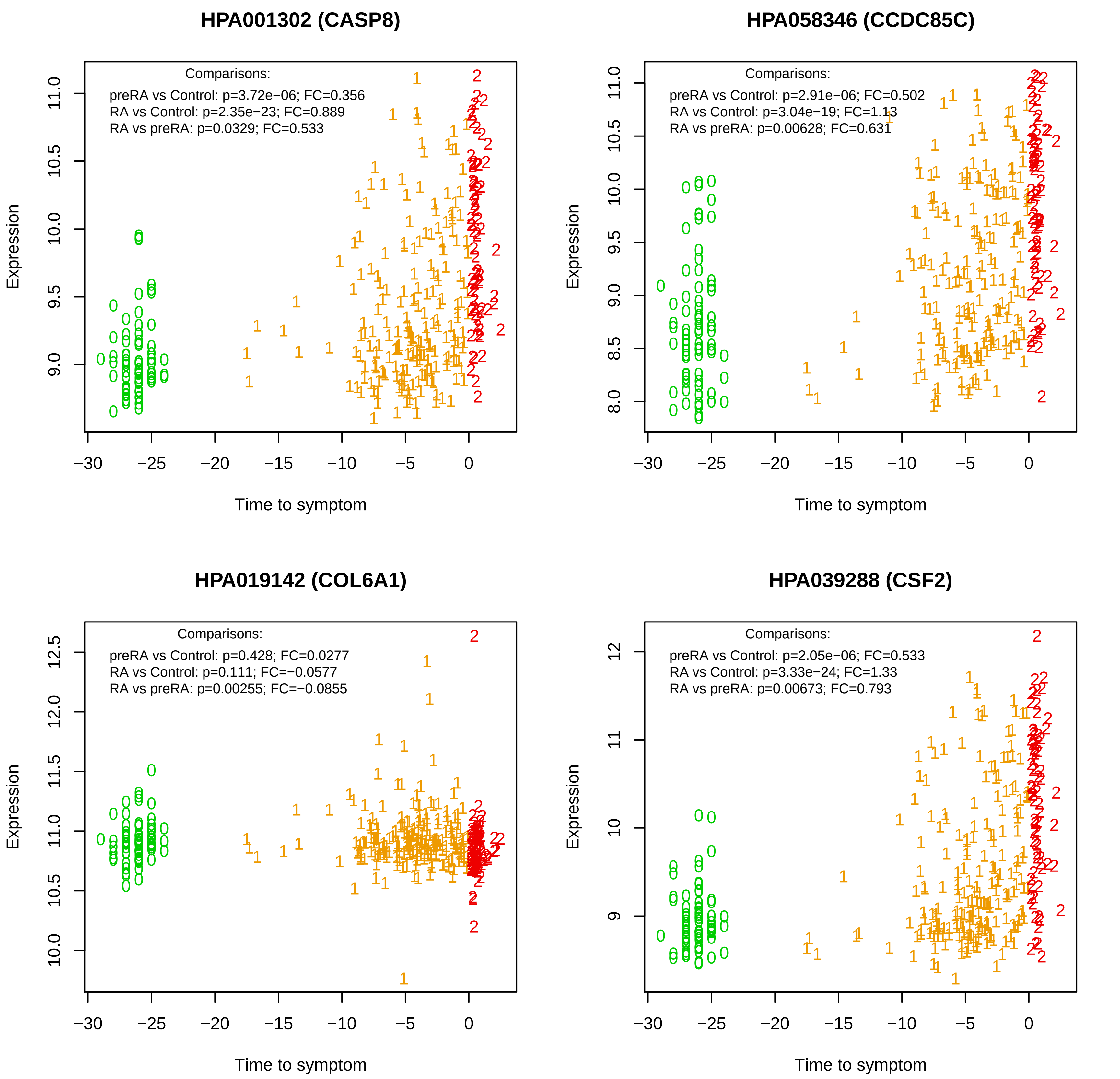
HPA001302 (CASP8)
Comparisons:
preRA vs Control: p=3.72e−06; FC=0.356
RA vs Control: p=2.35e−23; FC=0.889
RA vs preRA: p=0.0329; FC=0.533
Expression
Time to symptom
HPA058346 (CCDC85C)
Comparisons:
preRA vs Control: p=2.91e−06; FC=0.502
RA vs Control: p=3.04e−19; FC=1.13
RA vs preRA: p=0.00628; FC=0.631
Expression
Time to symptom
HPA019142 (COL6A1)
Comparisons:
preRA vs Control: p=0.428; FC=0.0277
RA vs Control: p=0.111; FC=−0.0577
RA vs preRA: p=0.00255; FC=−0.0855
Expression
Time to symptom
HPA039288 (CSF2)
Comparisons:
preRA vs Control: p=2.05e−06; FC=0.533
RA vs Control: p=3.33e−24; FC=1.33
RA vs preRA: p=0.00673; FC=0.793
Expression
Time to symptom

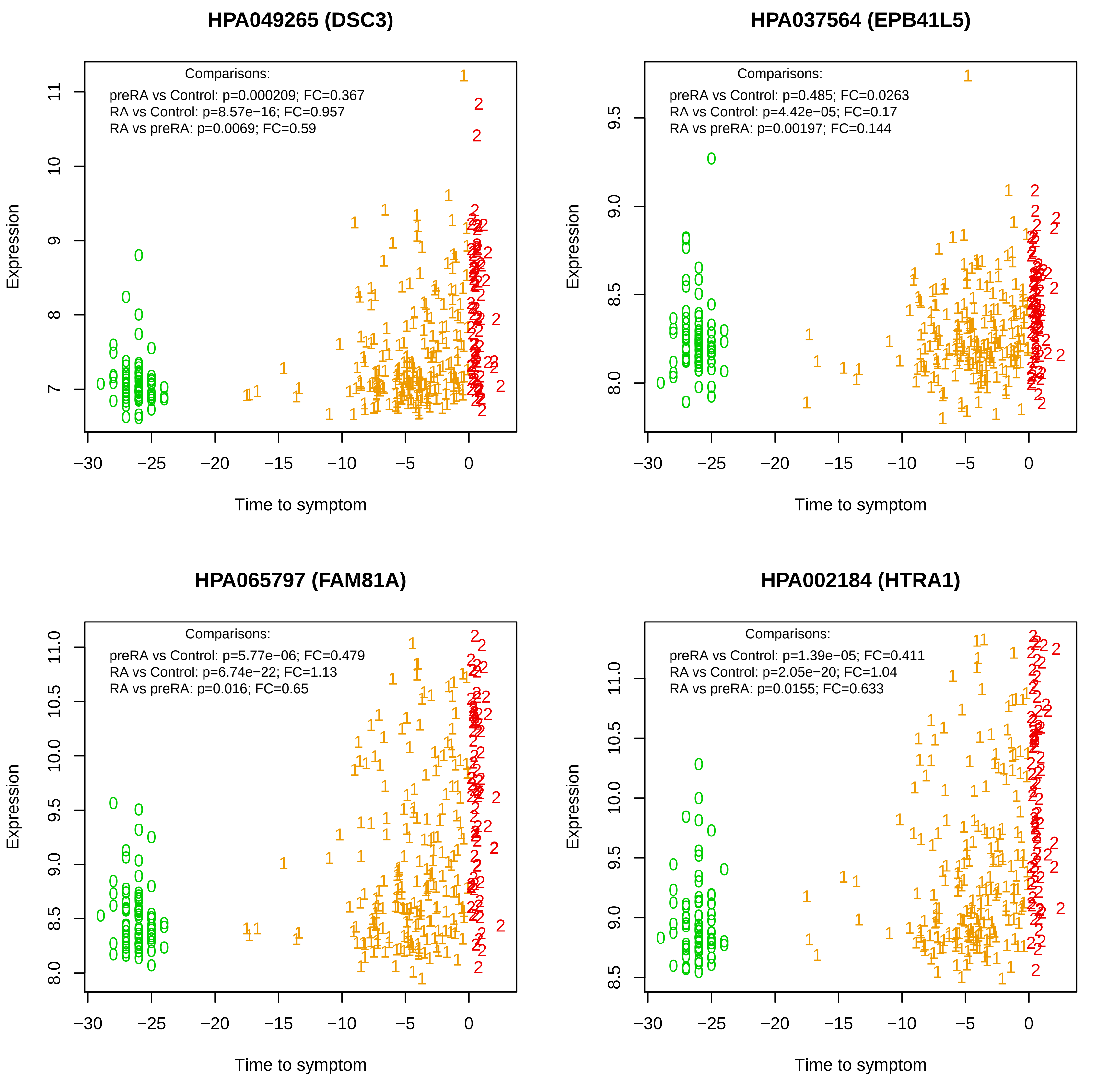
HPA049265 (DSC3)
Comparisons:
preRA vs Control: p=0.000209; FC=0.367
RA vs Control: p=8.57e−16; FC=0.957
RA vs preRA: p=0.0069; FC=0.59
Expression
Time to symptom
HPA037564 (EPB41L5)
Comparisons:
preRA vs Control: p=0.485; FC=0.0263
RA vs Control: p=4.42e−05; FC=0.17
RA vs preRA: p=0.00197; FC=0.144
Expression
Time to symptom
HPA065797 (FAM81A)
Comparisons:
preRA vs Control: p=5.77e−06; FC=0.479
RA vs Control: p=6.74e−22; FC=1.13
RA vs preRA: p=0.016; FC=0.65
Expression
Time to symptom
HPA002184 (HTRA1)
Comparisons:
preRA vs Control: p=1.39e−05; FC=0.411
RA vs Control: p=2.05e−20; FC=1.04
RA vs preRA: p=0.0155; FC=0.633
Expression
Time to symptom

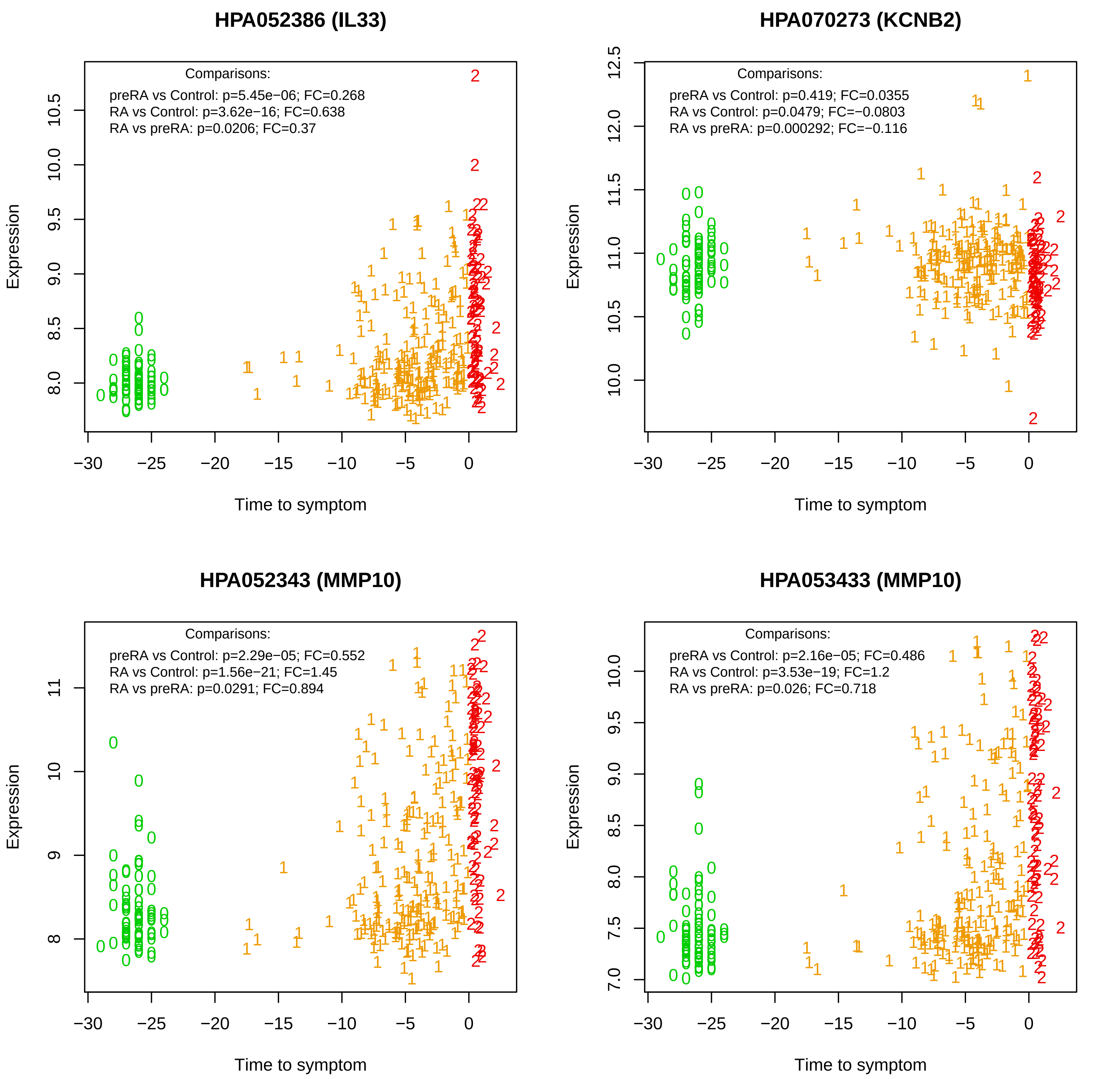
HPA052386 (IL33)
Comparisons:
preRA vs Control: p=5.45e−06; FC=0.268
RA vs Control: p=3.62e−16; FC=0.638
RA vs preRA: p=0.0206; FC=0.37
Expression
Time to symptom
HPA070273 (KCNB2)
Comparisons:
preRA vs Control: p=0.419; FC=0.0355
RA vs Control: p=0.0479; FC=−0.0803
RA vs preRA: p=0.000292; FC=−0.116
Expression
Time to symptom
HPA052343 (MMP10)
Comparisons:
preRA vs Control: p=2.29e−05; FC=0.552
RA vs Control: p=1.56e−21; FC=1.45
RA vs preRA: p=0.0291; FC=0.894
Expression
Time to symptom
HPA053433 (MMP10)
Comparisons:
preRA vs Control: p=2.16e−05; FC=0.486
RA vs Control: p=3.53e−19; FC=1.2
RA vs preRA: p=0.026; FC=0.718
Expression
Time to symptom

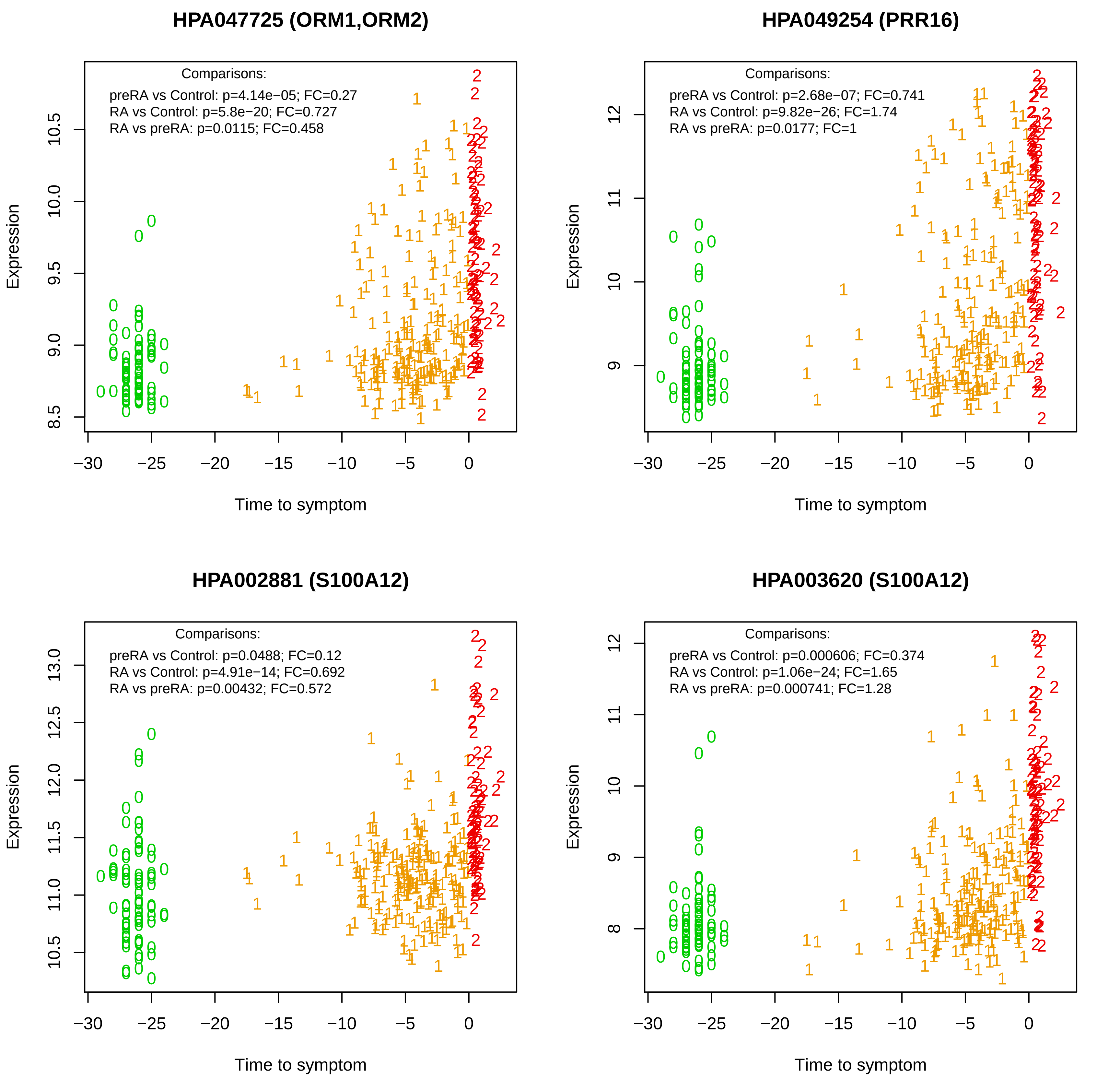
HPA047725 (ORM1,ORM2)
Comparisons:
preRA vs Control: p=4.14e−05; FC=0.27
RA vs Control: p=5.8e−20; FC=0.727
RA vs preRA: p=0.0115; FC=0.458
Expression
Time to symptom
HPA049254 (PRR16)
Comparisons:
preRA vs Control: p=2.68e−07; FC=0.741
RA vs Control: p=9.82e−26; FC=1.74
RA vs preRA: p=0.0177; FC=1
Expression
Time to symptom
HPA002881 (S100A12)
Comparisons:
preRA vs Control: p=0.0488; FC=0.12
RA vs Control: p=4.91e−14; FC=0.692
RA vs preRA: p=0.00432; FC=0.572
Expression
Time to symptom
HPA003620 (S100A12)
Comparisons:
preRA vs Control: p=0.000606; FC=0.374
RA vs Control: p=1.06e−24; FC=1.65
RA vs preRA: p=0.000741; FC=1.28
Expression
Time to symptom

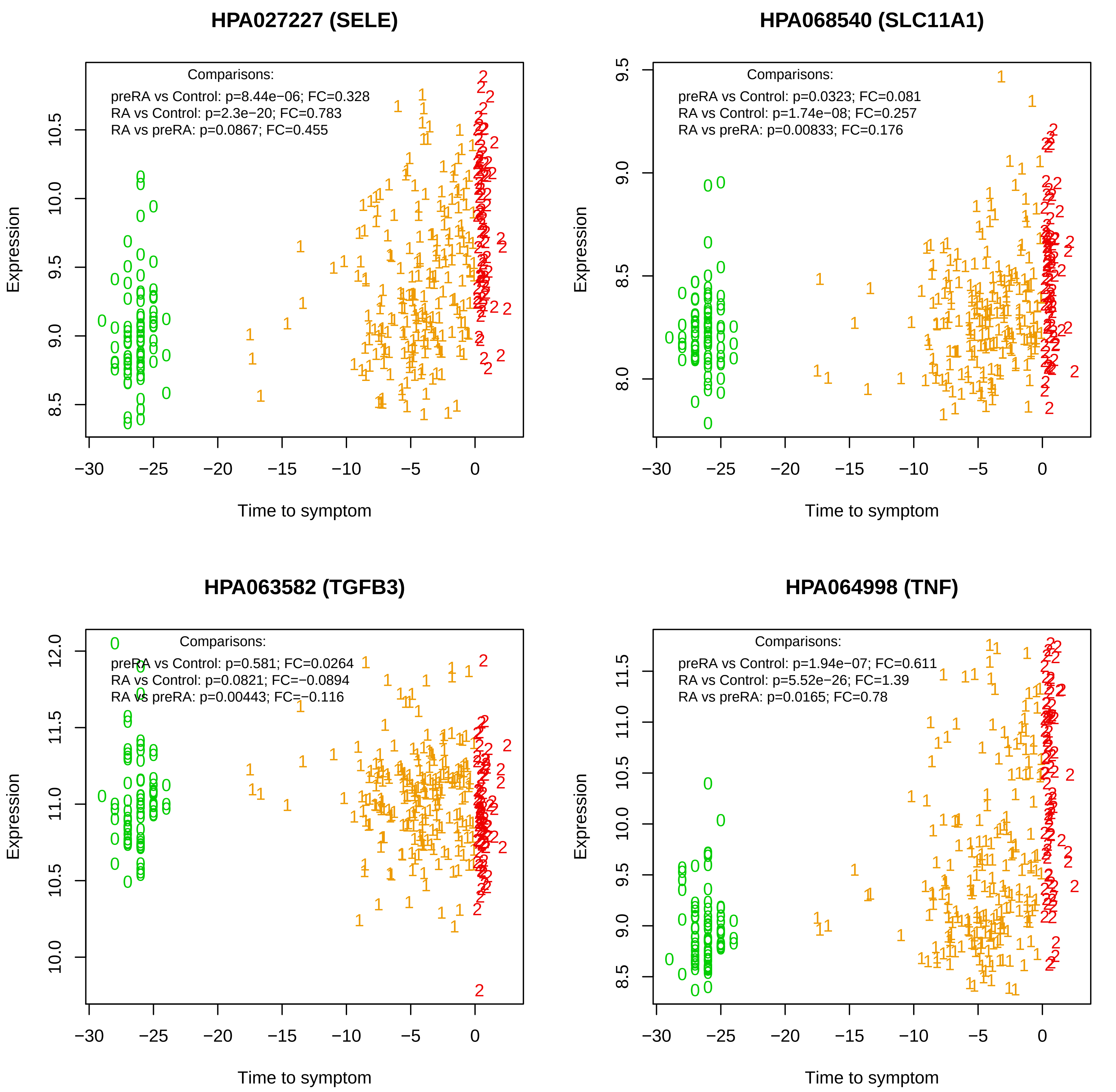
HPA027227 (SELE)
Comparisons:
preRA vs Control: p=8.44e−06; FC=0.328
RA vs Control: p=2.3e−20; FC=0.783
RA vs preRA: p=0.0867; FC=0.455
Expression
Time to symptom
HPA068540 (SLC11A1)
Comparisons:
preRA vs Control: p=0.0323; FC=0.081
RA vs Control: p=1.74e−08; FC=0.257
RA vs preRA: p=0.00833; FC=0.176
Expression
Time to symptom
HPA063582 (TGFB3)
Comparisons:
preRA vs Control: p=0.581; FC=0.0264
RA vs Control: p=0.0821; FC=−0.0894
RA vs preRA: p=0.00443; FC=−0.116
Expression
Time to symptom
HPA064998 (TNF)
Comparisons:
preRA vs Control: p=1.94e−07; FC=0.611
RA vs Control: p=5.52e−26; FC=1.39
RA vs preRA: p=0.0165; FC=0.78
Expression
Time to symptom

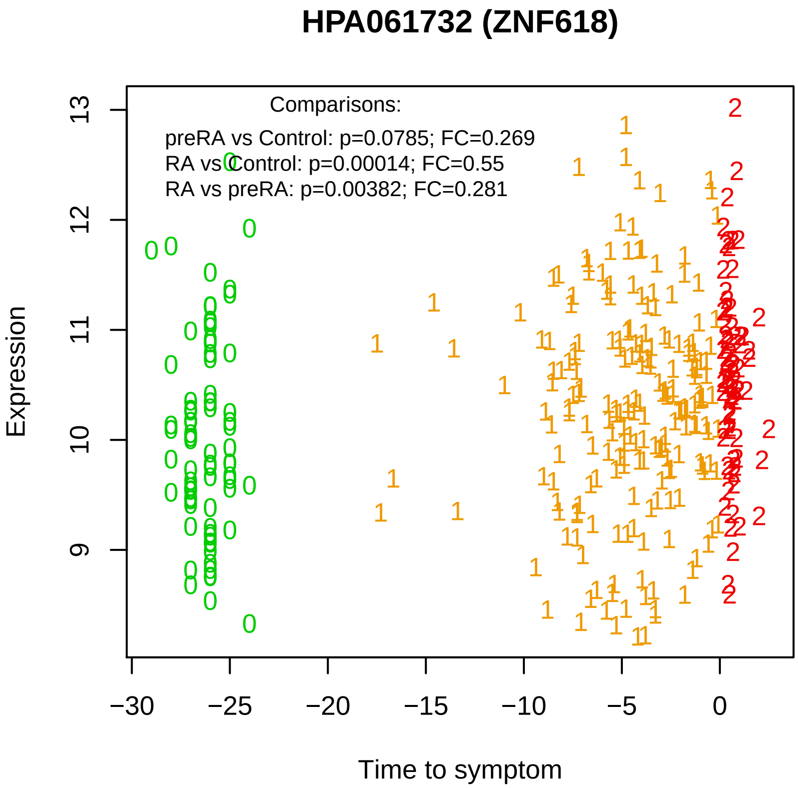
HPA061732 (ZNF618)
Comparisons:
preRA vs Control: p=0.0785; FC=0.269
RA vs Control: p=0.00014; FC=0.55
RA vs preRA: p=0.00382; FC=0.281
Expression
Time to symptom
13
12
11
10
9
−30
−25
−20
−15
−10
−5
0
